# Core histones govern echinocandin susceptibility in *Candida glabrata*

**DOI:** 10.1128/spectrum.02399-24

**Published:** 2025-04-30

**Authors:** Aditi Pareek, Rupinder Kaur

**Affiliations:** 1 Laboratory of Fungal Pathogenesis, Centre for DNA Fingerprinting and Diagnostics 28627, Hyderabad, India; 2 Graduate studies, Manipal Academy of Higher Education, Manipal, Karnataka, India; Institut Pasteur, Paris, France

**Keywords:** histones, linker histone, histone chaperone, azoles, echinocandins, reactive oxygen species, H3K56 acetylation

## Abstract

The dynamic chromatin structure regulates many biological processes including gene expression, DNA repair, and genome stability in eukaryotic cells. However, its role in governing antifungal drug susceptibility in medically important fungi is just beginning to be deciphered. Chromatin architecture is maintained by a complex interplay among histone protein stoichiometry sustainment, post-translational modifications of histone proteins, and the activity of chromatin remodeling complexes. Herein, we report that the reduced gene dosage of histone core proteins in the opportunistic human fungal pathogen *Candida glabrata* leads to increased susceptibility toward the widely used, cell wall-targeting echinocandin antifungal drugs. Our comprehensive characterization of single and double histone mutants revealed that linker histone H1 loss had no effect on cell physiology and drug susceptibility, whereas low H2A, H2B, H3, and H4 protein levels resulted in decreased reactive oxygen species production, altered biofilm production, elevated DNA damage, and echinocandin stress susceptibility. Importantly, not all core histone mutants exhibited an increased sensitivity to other cell wall stressors, thereby precluding a general cell wall defect accounting solely for the increased caspofungin susceptibility. Finally, we show that the histone H3 acetylation at lysine-56 may be pivotal to caspofungin response of *C. glabrata*, as H3K56Ac levels were reduced in both core histone mutants and upon caspofungin exposure, with H3K56 acetyltransferase (CgRtt109)- and nucleosome assembly factor (CgAsf1)-lacking mutants displaying increased caspofungin susceptibility. Besides demonstrating the histone requirement for the survival of *C. glabrata* in the mouse systemic candidiasis model, our findings unveil histone dosage-regulated cellular processes that impact echinocandin susceptibility.

**IMPORTANCE:**

Echinocandin antifungals, which impede cell wall synthesis, are often used to treat Candida bloodstream infections. The human opportunistic fungal pathogen *Candida* (Nakaseomyces) *glabrata* is increasingly being reported to exhibit co-resistance to echinocandins and ergosterol biosynthesis-inhibitory azole drugs in hospitals worldwide. However, the role of histones, protein-building blocks of the nucleosome, in governing echinocandin resistance in *C. glabrata* is not understood. Herein, we show that the reduced gene dosage of core histone proteins, but not of the linker histone, leads to echinocandin susceptibility, which is partly due to increased ROS levels. Additionally, our data implicate histone H3 acetylation at lysine-56 in the caspofungin response of *C. glabrata*. Since the emerging echinocandin resistance is an impediment to successful antifungal therapy, our findings open up a new research avenue of pharmacological targeting of histone proteins that could potentially block echinocandin resistance and attenuate *C. glabrata* survival in the host.

## INTRODUCTION

A nucleosome, the basic recurring unit of the chromatin in eukaryotes, consists of ~146 bp DNA wrapped around an octamer of histone proteins (1, 2). The canonical histone proteins, which are conserved across eukaryotes, are represented by four core histones, H2A, H2B, H3, and H4, and the linker histone H1 (1, 2). Histones H2A and H2B form a heterodimer, whereas histones H3 and H4 form a tetramer, and the histone octamer, which is comprised of a H3-H4 tetramer flanked by two copies of the H2A-H2B dimer, forms the core of the nucleosome particle (1, 2). The linker histone H1, which binds to the nucleosome at the entry and exit sites of the internucleosomal DNA, is pivotal to the higher-order chromatin structure organization (2, 3). Histone proteins and their post-translational modifications play an important role in cellular stress responses across the eukaryotic kingdom (2, 4–6). Consistent with this, histone gene dosage alterations have been associated with reduced growth and virulence of human pathogenic fungi (7–9).

Histone H3 and H4 proteins are encoded by three ORFs in the opportunistic human fungal pathogen *Candida glabrata* (*Nakaseomyces glabratus*) (8, 10). *C. glabrata* causes superficial infections of the mucosal surfaces as well as invasive infections (10, 11). *C. glabrata* prevalence varies across different geographical regions, with *C. glabrata* bloodstream infections being associated with high mortality rates (12–14). The successful treatment of *C. glabrata* infections is hindered by its low inherent susceptibility to azole antifungal drugs and a high propensity to acquire azole and echinocandin antifungal resistance in hospitals worldwide (12, 13, 15). Although about 10% of *C. glabrata* isolates exhibit azole drug resistance in clinical settings, a rise in the number of *C. glabrata* isolates displaying resistance to both azole and echinocandin drugs is progressively being reported (12, 13, 15–18).

Azole and echinocandin antifungal drugs target the fungal cell membrane and the cell wall, respectively (10, 16). The fungistatic azole drugs impede *C. glabrata* growth by inhibiting lanosterol 14-α demethylase enzyme, encoded by the *CgERG11* gene (10, 16). The lanosterol demethylase enzyme is an important enzyme of the ergosterol biosynthesis pathway, catalyzes C-14 demethylation of lanosterol, and converts lanosterol to 4,4-dimethyl cholesta-8,14,24-triene-3-β-ol during ergosterol biosynthesis (10, 16, 19). The fungicidal echinocandin drugs bind to 1,3-β-glucan synthase enzyme, which uses UDP-Glc (uridine diphosphate-activated glucose) as a substrate and transfers glucose from UDP-glucose to the growing glucan chain by catalyzing the formation of β−1,3-glycosidic linkages in 1,3-β-glucan, followed by 1,3-β-glucan transport across the plasma membrane (10, 16, 20). β−1,3-glucan is a structural constituent of the *C. glabrata* cell wall, and its synthesis inhibition is associated with a compensatory increase in chitin levels in the fungal cell wall in some cases (10, 15). 1,3-β-glucan synthase enzyme is encoded by three genes, *CgFKS1*, *CgFKS2,* and *CgFKS3*, in *C. glabrata* (10, 16). Mutations in hot-spot regions of *CgFKS1* and *CgFKS2* genes primarily contribute to echinocandin resistance in hospital settings (10, 15, 16). Contrarily, the resistance to azole antifungals in clinical isolates of *C. glabrata* has largely been attributed to overexpression of multidrug transporters, arising mostly from gain-of-function mutations in *CgPDR1* gene that codes for a master regulator of azole transporter gene expression (10, 16).

Echinocandins constitute the first-line antifungal therapy for invasive *C. glabrata* infections, due to a high prevalence of azole resistance in *C. glabrata* (12, 13, 15). However, the emergence of co-resistance to azole and echinocandins poses a grave threat to the success of antifungal therapy against *C. glabrata* infections (12, 13, 16–18). Recent transcriptional and genome-wide mutational studies have unveiled the association of ergosterol biosynthesis genes with caspofungin susceptibility in *C. glabrata* (19, 21). Moreover, the contribution of cellular stress responses to echinocandin susceptibility has been reported, with echinocandin exposure leading to elevated reactive oxygen species (ROS) production, altered mitochondrial functions, and ergosterol biosynthesis gene downregulation (15, 21, 22). Importantly, the dysregulation of both ER stress-responsive calcineurin signaling and cell wall stress-responsive protein kinase C-mediated cell wall integrity pathway leads to increased susceptibility to azole as well as echinocandin drugs (10, 15), thereby highlighting the cross-talk between these two cellular signaling pathways for antifungal tolerance.

The accumulating evidence highlights the role of chromatin architecture in modulating virulence and antifungal drug resistance in *C. glabrata* (19, 23). It has been reported that enzymes involved in histone acetylation, deacetylation, methylation, and demethylation govern the susceptibility of *C. glabrata* toward azole and echinocandin drugs (19, 23). Additionally, loss of the ATPase subunit of the SWI/SNF chromatin remodeling complex, which governs nucleosome position on the chromatin, has recently been shown to result in increased azole susceptibility in *C. glabrata* (24).

The post-translational modifications of histone proteins are likely to be pivotal to antifungal resistance gene expression, as these regulate the accessibility of DNA to various transcriptional regulators (2, 19, 23). However, the effect of histone protein levels *per se* on cellular processes in antifungal-treated fungal cells remains to be investigated. Toward this end, through a comprehensive analysis of single and double histone mutants, we herein report that a decrease in the gene dosage of core histone proteins, but not of the linker histone, leads to caspofungin susceptibility in *C. glabrata*. Additionally, we show that although reduced histone H2A, H2B, H3, and H4 levels neither affected fluconazole susceptibility nor cell wall stress survival, these led to deficient biofilm formation and reduced survival in the mouse systemic candidiasis model. Finally, our data attribute the elevated caspofungin susceptibility of histone mutants partly to increased ROS levels and underscore the hitherto unknown roles of core histones in the pathobiology of *C. glabrata*.

## RESULTS

### Two ORFs code for histone H2A and H2B proteins in *C. glabrata*

To systematically investigate the effect of histone levels on drug susceptibility in *C. glabrata*, we generated deletion strains lacking genes coding for core histones, H2A and H2B, and the linker histone H1 (Table S1). Mutants with different dosages of histone H3 and H4 genes (8) were previously constructed in the laboratory. Notably, the histone H2A is encoded by two ORFs, *CgHTA1* (*CAGL0K11440g*) and *CgHTA2* (*CAGL0C04411g*) (Fig. S1A, Table S1 ), with protein products of these two ORFs differing only in one amino acid at the N-terminus (Fig. S1B) (25). Similarly, the histone H2B is encoded by two ORFs, *CgHTB1* (*CAGL0K11462g*) and *CgHTB2* (*CAGL0C04389g*) (Table S1), whose protein products differ from each other at one position, with CgHtb2 protein also being shorter by two amino acids, compared with CgHtb1 (Fig. S1C). Importantly, the genome architectures of *CgHTA1-CgHTB1* and *CgHTA2-CgHTB2* gene loci (Fig. S1A) are similar to their *Saccharomyces cerevisiae* counterparts (https://www.yeastgenome.org/). The linker histone in *C. glabrata* is encoded by a single gene *CgHHO1* (*CAGL0M02783g*) (Table S1), similar to its *S. cerevisiae* ortholog. Of note, histones H3 and H4 are encoded by three ORFs, *CgHHT1* (*CAGL0C04114g*)*, CgHHT2* (*CAGL0M06655g*) and *CgHHT3* (*CAGL0H09856g*), and *CgHHF1* (*CAGL0C04136g*), *CgHHF2* (*CAGL0M06677g*), and *CgHHF3* (*CAGL0H09834g*), respectively, in *C. glabrata* (Table S1), contrary to two histone H3 and histone H4 genes coding for H3 and H4 proteins, respectively, in *S. cerevisiae* (8).

### *CgHTA2* and *CgHTB2* genes contribute more to histone protein expression

**Fig 1:**
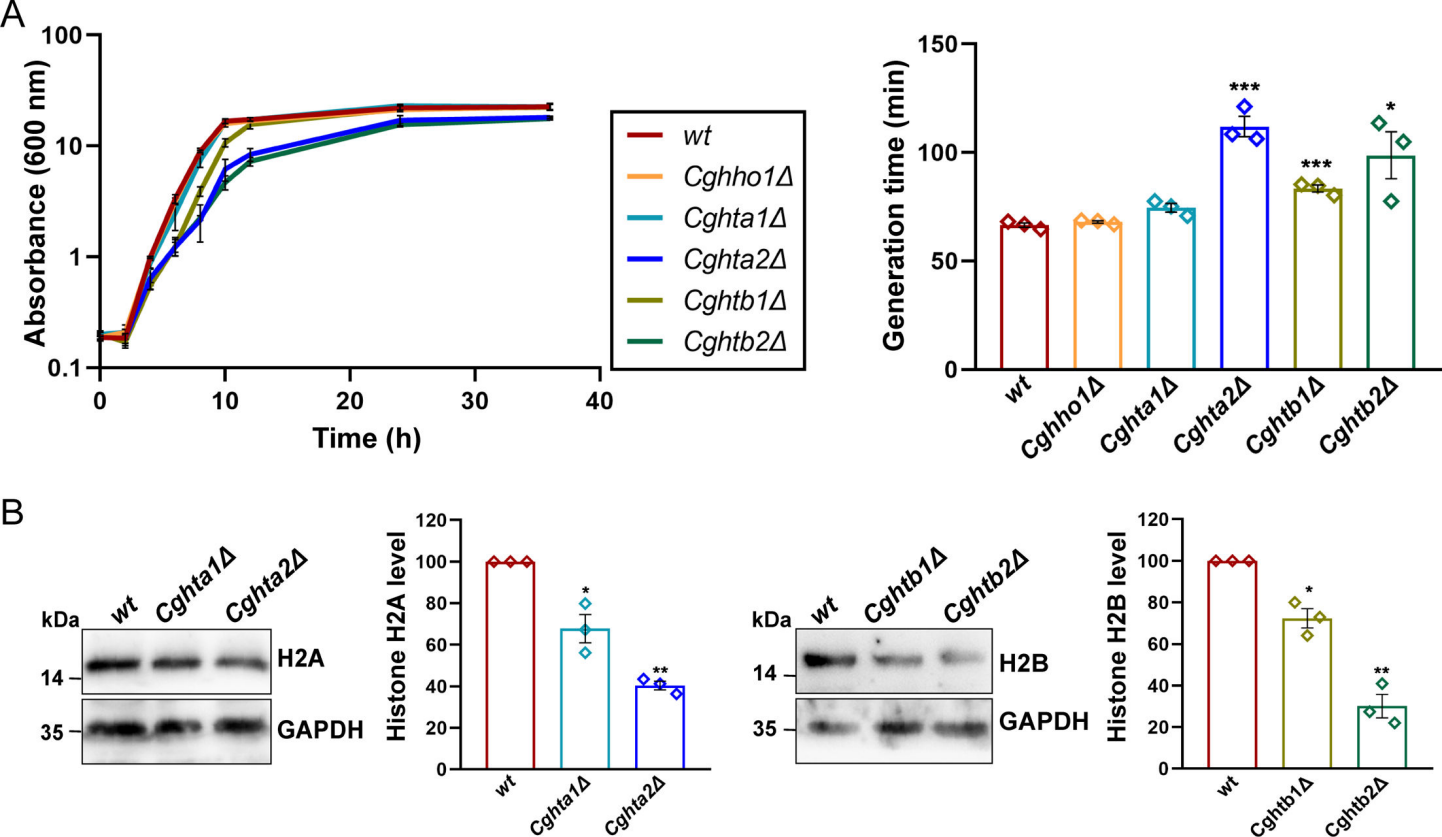
*CgHTA2* and *CgHTB2* gene loss leads to growth retardation in *C. glabrata*. (**A**) Growth curve analysis of indicated *C. glabrata* strains in YPD medium. Doubling time was calculated during the logarithmic phase of growth (2–8 h) and is indicated on the right side of the line graph. Data represent mean ± SEM (*n* = 3). *P ≤ 0.05; ***P ≤ 0.005; unpaired two-tailed Student’s *t* test. (**B**) Representative western blots showing histone H2A and H2B levels in indicated *C. glabrata* strains. Whole cell lysates (60 µg) were resolved on 15% SDS-PAGE and probed with anti*-*H2A, anti-H2B, and anti-GAPDH antibodies. For quantification, signal intensity in each lane was measured using the ImageJ software, and histone H2A and H2B levels were normalized against CgGapdh (used as loading control) signal for each strain. Data (mean ± SEM; *n* = 3) represent a change in histone levels in mutants, compared with *wt* (considered as 100). *P ≤ 0.05; **P ≤ 0.01; paired two-tailed Student’s *t* test.

After generating the deletion strains, *Cghho1Δ* (lacks the linker histone H1-encoding ORF), *Cghta1Δ* (lacks the histone H2A-encoding *CgHTA1* ORF), *Cghtb1Δ* (lacks the histone H2B-encoding *CgHTB1* ORF), *Cghta2Δ* (lacks the histone H2A-encoding *CgHTA2* ORF), and *Cghtb2Δ* (lacks the histone H2B-encoding *CgHTB2* ORF) (Table S1), we next checked if the reduced histone dosage affects cell growth. Notably, a lower histone H3 and H4 gene dosage was previously found to be linked with diminished growth potential (8). We found that *Cghta2Δ* and *Cghtb2Δ* mutants were slow growers and displayed ~50%^–^70% longer doubling time, compared to wild-type (*wt*) cells in the rich YPD medium (Fig. 1A). The time-course analysis also revealed that the loss of *CgHHO1* and *CgHTA1* genes had no effect on cell growth, whereas *CgHTB1* gene loss led to mildly impaired growth (Fig. 1A).

To determine the basis underlying differential growth profiles of mutants lacking histone H2A and H2B ORFs, we performed western analysis using anti-H2A and anti-H2B antibodies. We observed that *CgHTA2* and *CgHTB2* gene loss led to a 60% and 70% reduction in H2A and H2B levels, respectively (Fig. 1B). Contrarily, *CgHTA1* and *CgHTB1* gene disruption decreased H2A and H2B levels by ~30%, respectively (Fig. 1B). Together, these results suggest that under regular growth conditions, *CgHTA2* and *CgHTB2* genes contribute more to H2A and H2B protein production, respectively. These data also raise the possibility of attenuated growth of *Cghta2Δ* and *Cghtb2Δ* mutants due to highly diminished histone H2A and H2B levels, respectively. Besides underscoring the core histone requirement for cell growth, these results raise the possibility of differential regulation of histone ORFs. In accordance, of three H3 and H4-encoding ORFs in *C. glabrata*, *CgHHT1-3* and *CgHHF1-3*, respectively, *CgHHT2* and *CgHHF2* contributed more to histone H3 and H4 expression, respectively (8). Of note, we were unable to measure histone H1 levels in *Cghho1Δ* mutant, as the commercially available anti-histone H1 antibody failed to specifically recognize the CgHho1 protein.

### Histone H1, H2A, and H2B are required for biofilm formation

**Fig 2:**
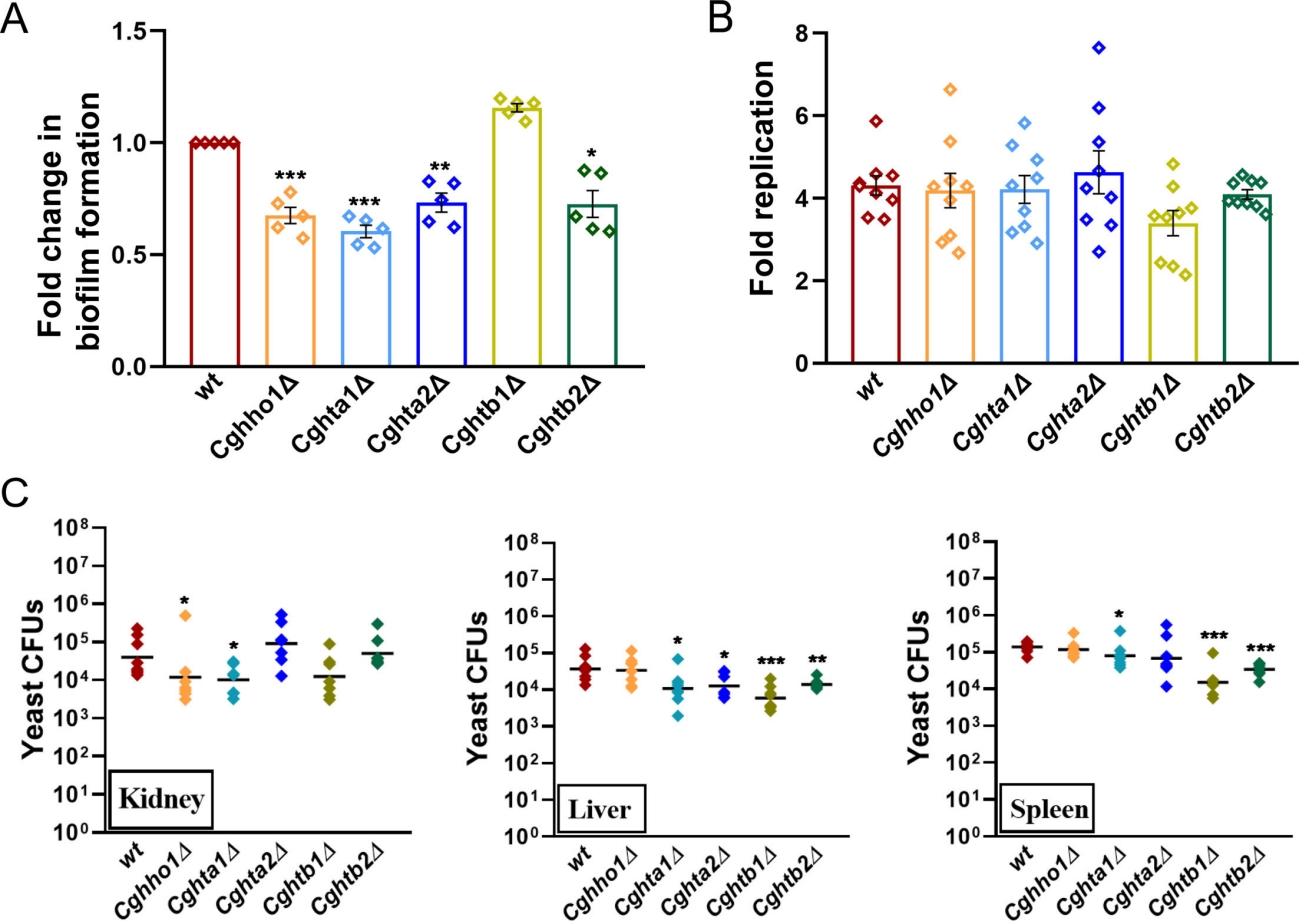
Histones H1, H2A, and H2B are required for survival of *C. glabrata* in mice. (**A**) Biofilm productivity of indicated *C. glabrata* strains in RPMI medium after 48 h incubation at 37^ο^C. Biofilm mass was determined from the absorbance values at 595 nm. Data (mean ± SEM; *n* = 5) represent a ratio of biofilm produced by mutant to that of *wt* (considered as 1.0). *P ≤ 0.05; **P ≤ 0.01; ***P ≤ 0.005; paired two-tailed Student’s *t* test. (**B**) *C. glabrata* survival analysis in human THP-1 macrophages. PMA-activated THP-1 cells were infected with indicated *C. glabrata* strains, followed by washing off the extracellular yeasts at 2 h and determining intracellular yeast load at 2 h and 24 h post-infection. Data (mean ± SEM; *n* = 9) represent fold replication of each strain which was calculated by dividing 24 h CFUs by 2 h CFUs. (**C**) CFU-based analysis of *C. glabrata* in the murine model of systemic candidiasis. Six to 8-week-old female BALB/c mice (*n* = 8) were infected with indicated *C*. *glabrata* strains (100 µL; 4 × 10^7^ cells), followed by mice sacrifice and organ collection at the seventh day post-infection. Kidneys, liver, and spleen were homogenized, and appropriate homogenate dilutions were plated on YPD medium supplemented with penicillin and streptomycin. *C. glabrata* CFUs, recovered from each organ, were calculated and plotted as an aligned dot plot. Each diamond and bar represent CFUs recovered from an individual mouse and CFU geometric mean, respectively. * *P* ≤ 0.05; **P ≤ 0.01; ***P ≤ 0.005; Mann-Whitney test.

Next, to investigate the consequences of histone H1, H2A, and H2B loss on virulence-associated traits, we checked the mutants’ ability to form biofilms on polystyrene, survive and replicate in macrophages, and survive in the murine model of systemic candidiasis. We found that although *Cghho1Δ*, *Cghta1Δ*, *Cghta2Δ,* and *Cghtb2Δ* mutants were deficient in biofilm formation, compared with *wt* cells (Fig. 2A), the *Cghtb1Δ* mutant displayed *wt*-like ability to form biofilms (Fig. 2A). Furthermore, all histone mutants proliferated like *wt* cells in human THP-1 macrophages (Fig. 2B). Histone mutants also exhibited lower fungal burden in BALB/c mice but in an organ-dependent manner (Fig. 2C). For example, the survival of *wt*, *Cghta2Δ*, *Cghtb1Δ,* and *Cghtb2Δ* strains was similar in kidneys, whereas the *Cghho1Δ* and *Cghta1Δ* CFUs (colony-forming units) were 3-fold to 4-fold lower in murine kidneys, compared with *wt* CFUs (Fig. 2C). Furthermore, compared with the *wt*-infected mice, the core histone mutant-infected mice contained less fungal burden in the liver, with *Cghta1Δ*, *Cghtb1Δ,* and *Cghtb2Δ* mutant-infected mice exhibiting reduced fungal burden in the spleen as well (Fig. 2C). Of note, CFUs recovered from the liver and the spleen of *Cghho1Δ*-infected and *wt*-infected mice were similar (Fig. 2C), suggesting that the linker histone is not important for survival of *C. glabrata* in the murine liver and spleen. Of note, although kidneys are the major target, the organ-dependent fungal burden of *C. glabrata* histone mutants in the systemic candidiasis model could be due to the organ-specific immune response including recruitment of specific cell types, microenvironment, metabolic state, nutrient availability, as well as the mouse organ-specific colonization potential of different histone mutants. Nevertheless, these data collectively implicate the linker histone H1 and the two core histones, H2A and H2B, in the survival of *C. glabrata* in the mammalian host.

### The loss of core histone proteins renders *C. glabrata* cells susceptible to caspofungin

**Fig 3:**
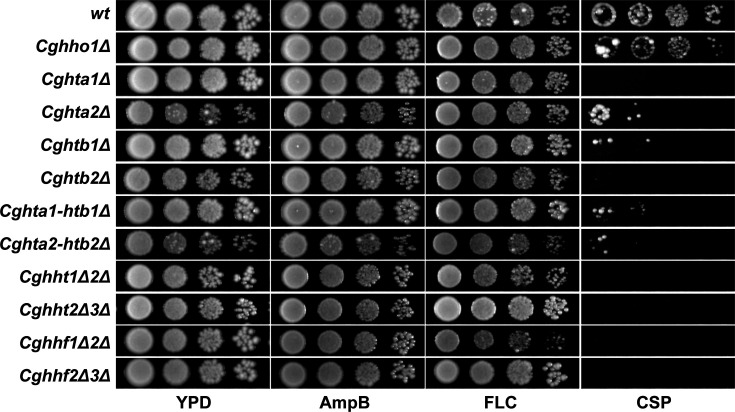
Core histones are essential for the survival of caspofungin stress in *C. glabrata*. Serial dilution spot assay illustrating the growth of indicated *C. glabrata* strains in YPD and YPD medium containing amphotericin B (AMB; 1 µg/mL), fluconazole (FLC; 16 µg/mL), and caspofungin (CSP; 150 ng/mL).

Next, we sought to investigate the histone protein requirement for antifungal drug tolerance. For this, we monitored the growth of *Cghho1Δ*, *Cghta1Δ*, *Cghta2Δ*, *Cghtb1Δ,* and *Cghtb2Δ* mutants as well as mutants with reduced histone H3 (*Cghht1Δ2Δ* and *Cghht2Δ3Δ*) and H4 (*Cghhf1Δ2Δ* and *Cghhf2Δ3Δ*) gene dosage in the medium containing fluconazole (azole drug), caspofungin (echinocandin drug), and amphotericin B (polyene antifungal). Surprisingly, compared with the *wt* strain, all core histone mutants exhibited exquisite sensitivity to caspofungin, whereas they grew similar to *wt* cells in the presence of fluconazole and amphotericin B (Fig. 3). Of note, the linker histone-lacking *Cghho1Δ* mutant displayed no growth defect in the medium containing caspofungin (Fig. 3). We also created a paired deletion of *CgHTA1-CgHTB1* and *CgHTA2-CgHTB2* genes and found the double mutants, *Cghta1Δ-htb1Δ* and *Cghta2Δ-htb2Δ*, to be equally sensitive to caspofungin (Fig. 3). These data point toward a unique role for core histones, H2A, H2B, H3, and H4, in regulating caspofungin susceptibility. The elevated caspofungin susceptibility of core histone mutants was verified by caspofungin dose-response assays wherein, compared with the *wt* and *Cghho1Δ* strains, the core histone mutants displayed a rapid decrease in OD_530_ (an indicator of growth inhibition) with increasing concentrations of caspofungin (Fig. S2). Furthermore, although attenuated growth of the core histone mutants in the presence of caspofungin (Fig. S2) is likely to be due to diminished core histone levels, strong growth-inhibitory effects of a high dose of caspofungin cannot be ruled out.

Since a decrease in histone H3 and H4 gene dosage has previously been linked with altered cell wall, thermal and DNA damage stress susceptibility, and/or unstable colony morphology in *Candida* (7–9), we next profiled the stress susceptibility of *C. glabrata* mutants carrying reduced levels of core and linker histone proteins. For this, we examined the growth of histone mutants in the medium containing the reductive stressor β-mercaptoethanol (β-ME), membrane-damaging agent SDS (Sodium dodecyl sulfate), cell wall stressors congo red (CR) and calcofluor white (CFW), oxidative stress-causing agents menadione and hydrogen peroxide (H_2_O_2_), DNA replication inhibitor hydroxyurea (HU), and DNA damage-causing agents, MMS (Methyl methanesulfonate), and phleomycin (Fig. S3). We found *Cghta2Δ*, *Cghtb2Δ, Cghta2Δ-htb2Δ,* and *Cghht2Δ3Δ* mutants to be uniquely highly sensitive to phleomycin, whereas the *Cghta1Δ-htb1Δ* mutant exhibited mild sensitivity to phleomycin and MMS (Fig. S3). Of note, phleomycin intercalates with the DNA and causes DNA strand breaks (26). Additionally, the simultaneous disruption of yeast cell wall proteins Cwp1 and Cwp2 is known to result in enhanced killing by phleomycin (27). Therefore, the elevated phleomycin susceptibility of *Cghta2Δ*, *Cghtb2Δ, Cghta2Δ-htb2Δ,* and *Cghht2Δ3Δ* mutants (Fig. S3) may be due to excessive DNA damage, impaired DNA repair, and/or higher influx of phleomycin owing to a weakened cell wall in these mutants.

**Fig 4:**
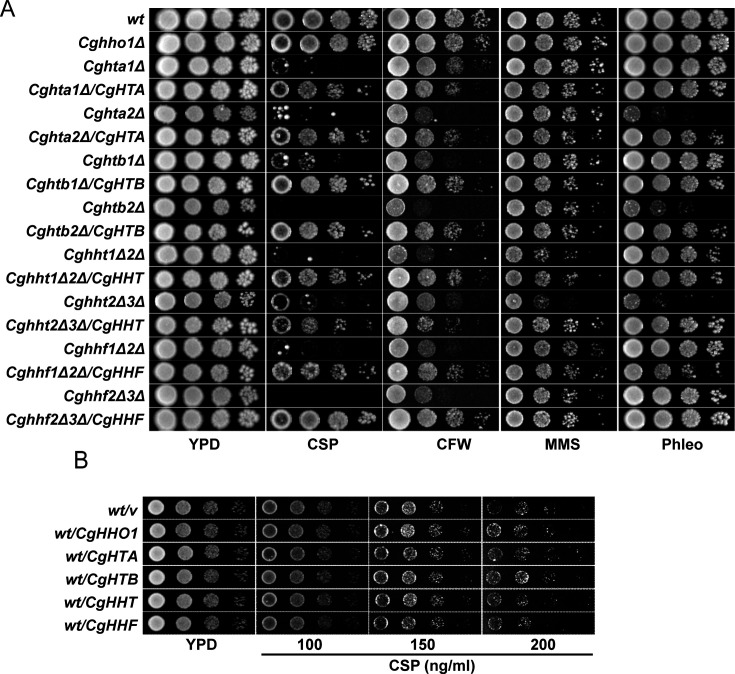
Histone gene overexpression has no effect on caspofungin susceptibility of *C. glabrata*. (**A**) Serial dilution spot assay illustrating the growth of indicated *C. glabrata* strains in YPD and YPD medium containing 150 ng/mL caspofungin (CSP), 2 mg/mL calcofluor white (CFW), 0.04% methylmethanesulfonate (MMS) and 10 µg/mL phleomycin (Phleo). (**B**) Serial dilution spot assay illustrating growth of indicated *C. glabrata* strains in YPD and YPD medium containing indicated concentrations of caspofungin (CSP).

Furthermore, as reported previously (8), *Cghht1Δ2Δ* and *Cghht2Δ3Δ* mutants were found to be sensitive to MMS-induced DNA damage (Fig. S3). Importantly, *Cghtb2Δ, Cghta1Δ-htb1Δ,* and *Cghta2Δ-htb2Δ* mutants displayed attenuated growth in the presence of the cell wall stressor CFW, but not CR (Fig. S3). Of note, the core histone mutants displayed differential sensitivity to other stresses including DNA damage (Fig.S3). These data suggest that the increased sensitivity of core histone mutants toward caspofungin is unlikely to be solely due to a general cell wall defect. Furthermore, ectopic expression of the histone genes, *CgHTA1*, *CgHTB1*, *CgHTA2*, *CgHTB2*, *CgHHF*, and *CgHHT*, rescued the reduced growth of respective histone mutants in the medium containing caspofungin, calcofluor white, methyl methanesulfonate, and phleomycin (Fig. 4A), indicating that the elevated susceptibility of the core histone mutants towards these stressors is due to reduced histone levels.

### Core histone levels are diminished upon caspofungin exposure

To delineate the mechanism/s underlying the caspofungin susceptibility of core histone mutants, we performed two experiments. First, we checked if histone gene overexpression affects caspofungin tolerance. For this, we expressed *CgHHO1*, *CgHTA*, *CgHTB*, *CgHHT,* and *CgHHF* genes from the strong *PDC1* promoter in *wt* cells and examined growth in the absence and presence of caspofungin. We observed no growth differences between these two conditions (Fig. 4B), indicating that increased histone levels did not lead to caspofungin tolerance (Fig. 4B).

**Fig 5:**
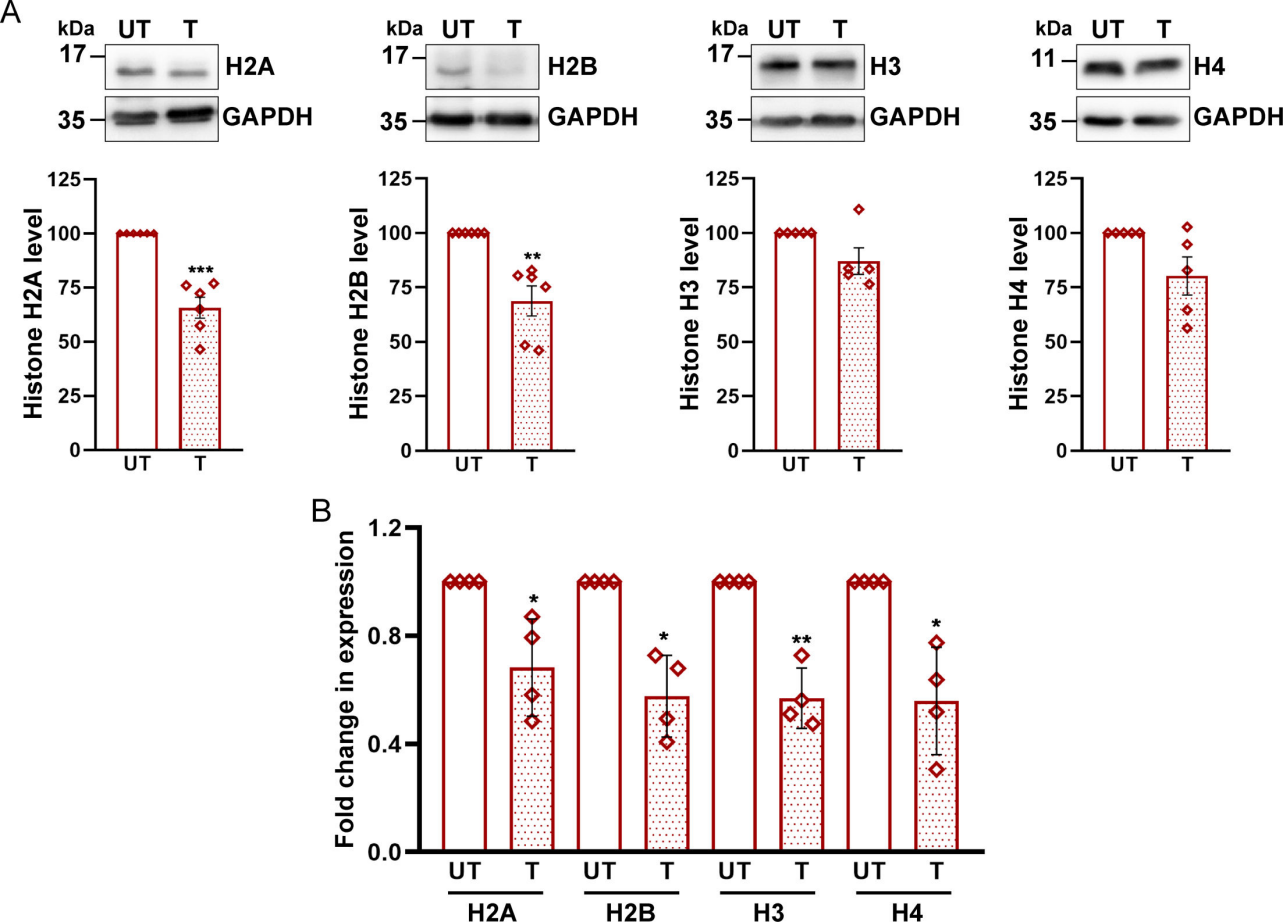
Caspofungin treatment leads to a reduction in core histone levels. (**A**) Representative western blots illustrating histone H2A, H2B, H3, and H4 levels in log phase cells of the *wild-type* (*wt*) *C. glabrata* strain. Cells were either treated (**T**) with caspofungin (200 ng/mL) for 1 h or left untreated (UT). Whole cell lysates (60 µg) were resolved on 15% SDS-PAGE and probed with indicated antibodies. For quantification, signal intensity in each lane was measured using the ImageJ software, and histone levels were normalized against CgGapdh (used as loading control) signal for each condition. Data (mean ± SEM; *n* = 5–6) represent a change in histone levels in caspofungin-treated *wt* cells, compared with untreated *wt* cells (considered as 100). **P ≤ 0.01; ***P ≤ 0.005; paired two-tailed Student’s *t* test. (**B**) qRT-PCR-based analysis of histone gene transcript levels in log-phase caspofungin-treated (200 ng/mL for 1 h) *wt* cells. Total RNA was extracted from *C. glabrata* cells, followed by cDNA synthesis and quantitative real-time reverse transcriptase PCR (qRT-PCR) using the SYBR-Green mix. Transcript levels of histone H2A (*CgHTA*), H2B (*CgHTB*), H3 (*CgHHT*), and H4 (*CgHHF*) genes were measured by the 2^-ΔΔ^C_T_ method, using *CgACT1* gene expression as a control. Data (mean ± SEM; *n* = 4) represent change in histone gene expression in caspofungin-treated (**T**) cells, compared with untreated cells (UT; taken as 1.0). * *P* ≤ 0.05; **P ≤ 0.01; paired two-tailed Student’s *t* test.

Second, we checked if histone protein levels are altered upon caspofungin exposure. Although we observed about a 35% reduction in H2A and H2B levels in caspofungin-treated *wt* cells, compared with untreated *wt* cells, changes in histone H3 and H4 were modest and not statistically significant (Fig. 5A). Importantly, caspofungin treatment lowered the histone H2A, H2B, H3, and H4 transcript levels by 50% (Fig. 5B), suggesting that *C. glabrata* responds to caspofungin exposure by downregulating the histone gene expression. In spite of decreased H3 and H4 transcript levels, a major change in H3 and H4 protein levels was not observed; the reason for this discrepancy is not clear and warrants further investigation. Since all core histone mutants displayed a similarly elevated susceptibility to caspofungin, we selected *Cghta2Δ*, *Cghtb2Δ*, *Cghht2Δ3Δ,* and *Cghhf2Δ3Δ* mutants for further analysis, as these mutants contained the least amount of histone H2A, H2B, H3, and H4 proteins, respectively [Fig. 1B; (8)].

### Elevated ROS production contributes to caspofungin susceptibility of the core histone mutants

**Fig 6:**
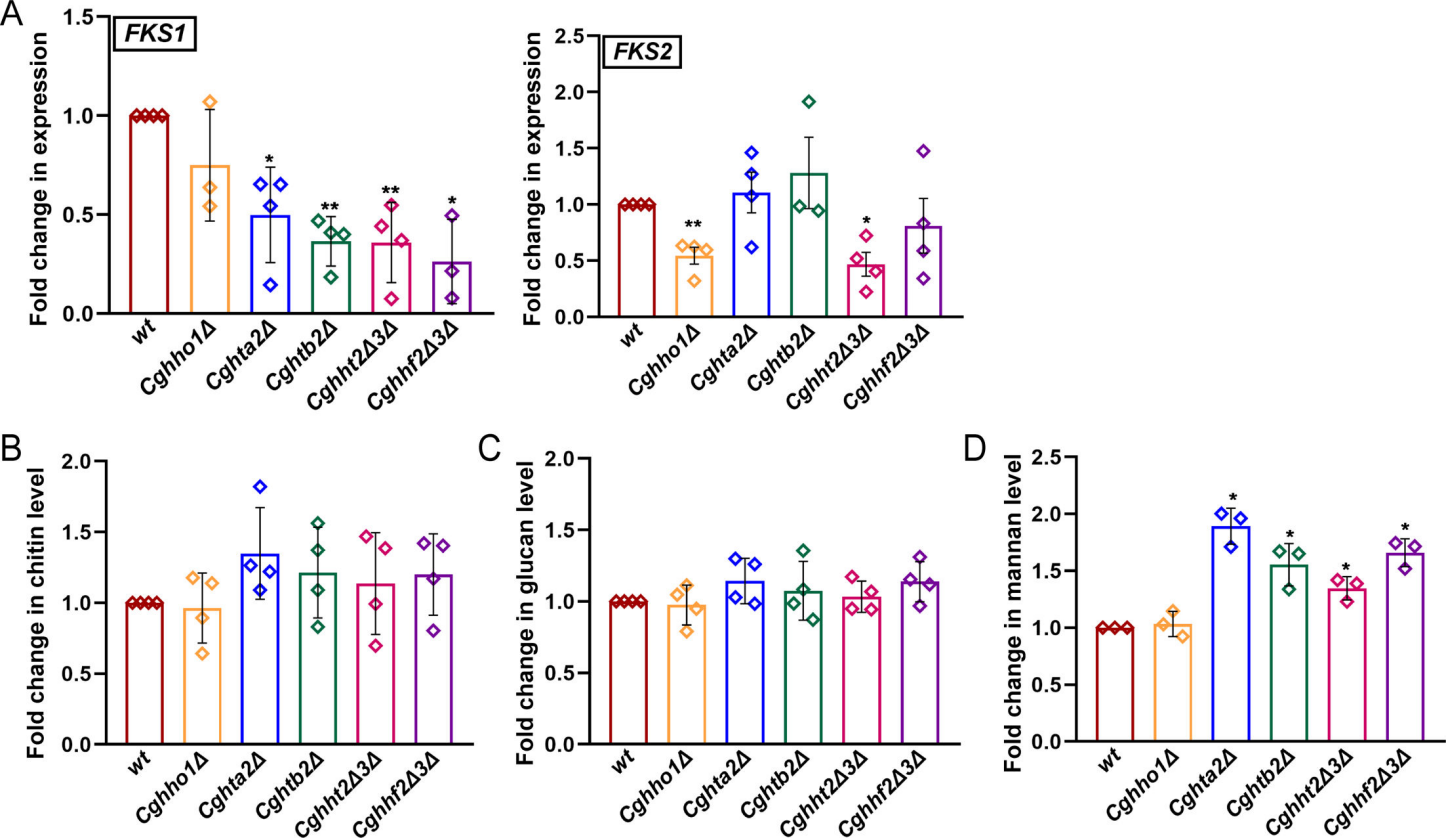
*CgFKS1* transcript levels are reduced upon reduction in the core histone gene dosage. (**A**) qRT-PCR-based analysis of *CgFKS1* and *CgFKS2* transcript levels in log-phase cultures of indicated *C. glabrata* strains. Total RNA extraction was followed by cDNA synthesis and qRT-PCR using the SYBR-Green mix. Transcript levels of *CgFKS1* and *CgFKS2* genes were measured by the 2^-ΔΔ^C_T_ method, using *CgACT1* gene expression as control. Data (mean ± SEM; *n* = 3–4) represent changes in *CgFKS1* and *CgFKS2* gene expression in mutants, compared with the wild-type (*wt*) strain (taken as 1.0). * *P* ≤ 0.05; **P ≤ 0.01; paired two-tailed Student’s *t* test. (**B**) Cell wall composition analysis. Log-phase cells (2.0 OD_600_) of indicated *C. glabrata* strains were collected and washed with PBS. After 30 min incubation at room temperature with calcofluor white, aniline blue, or FITC-conjugated Concanavalin A, for the measurement of chitin, glucan, and mannan, respectively, cells were washed thrice with PBS and analyzed using the flow cytometer. Data (mean ± SEM; *n* = 3–4) represent fold change in the levels of chitin, glucan, and mannan in the mutants, compared with the *wt* strain (taken as 1.0). *P ≤ 0.05; paired two-tailed Student’s *t* test.

All mutants with reduced histone gene dosage did not exhibit an increased sensitivity to cell wall stressors (Fig. S3). Since echinocandins target CgFks enzymes (10, 16), we next measured the expression of *CgFKS1* and *CgFKS2* genes in histone mutants. We found *CgFKS1* transcript levels to be 2-fold to 4-fold lower in the core histone mutants, compared with the *wt* strain (Fig. 6A). Furthermore, although no appreciable change in *CgFKS2* transcript levels was observed in *Cghta2Δ*, *Cghtb2Δ,* and *Cghhf2Δ3Δ* mutants, *CgFKS2* gene expression was 2-fold lower in *Cghho1Δ* and *Cghht2Δ3Δ* mutants (Fig. 6A). The molecular basis of reduced *CgFKS2* gene expression in *Cghho1Δ* and *Cghht2Δ3Δ* mutants is yet to be determined.

Next, to determine if reduced *CgFKS1* transcription leads to lower β-glucan in the cell wall of the core histone mutants, we measured levels of β-glucan, chitin, and mannan by aniline blue, calcofluor white, and FITC-conjugated concanavalin A-based staining assays, respectively, followed by the FACS-based fluorescence measurement. We found mannan levels to be higher in the core histone mutants but not in the linker histone mutant, compared with the *wt* strain (Fig. 6B). Chitin and β-glucan contents were similar in all strains (Fig. 6B), suggesting that the diminished *CgFKS1* gene expression in the core histone mutants does not translate into the reduced cell wall β-glucan. Consistent with this, *Cghta2Δ*, *Cghtb2Δ*, *Cghht2Δ3Δ,* and *Cghhf2Δ3Δ* mutants also displayed no elevated susceptibility to zymolyase (hydrolyzes β−1,3-glucan in the cell wall) treatment (Fig. S4), suggesting that the cell wall may not be substantially altered upon reduction in core histone levels.

**Fig 7:**
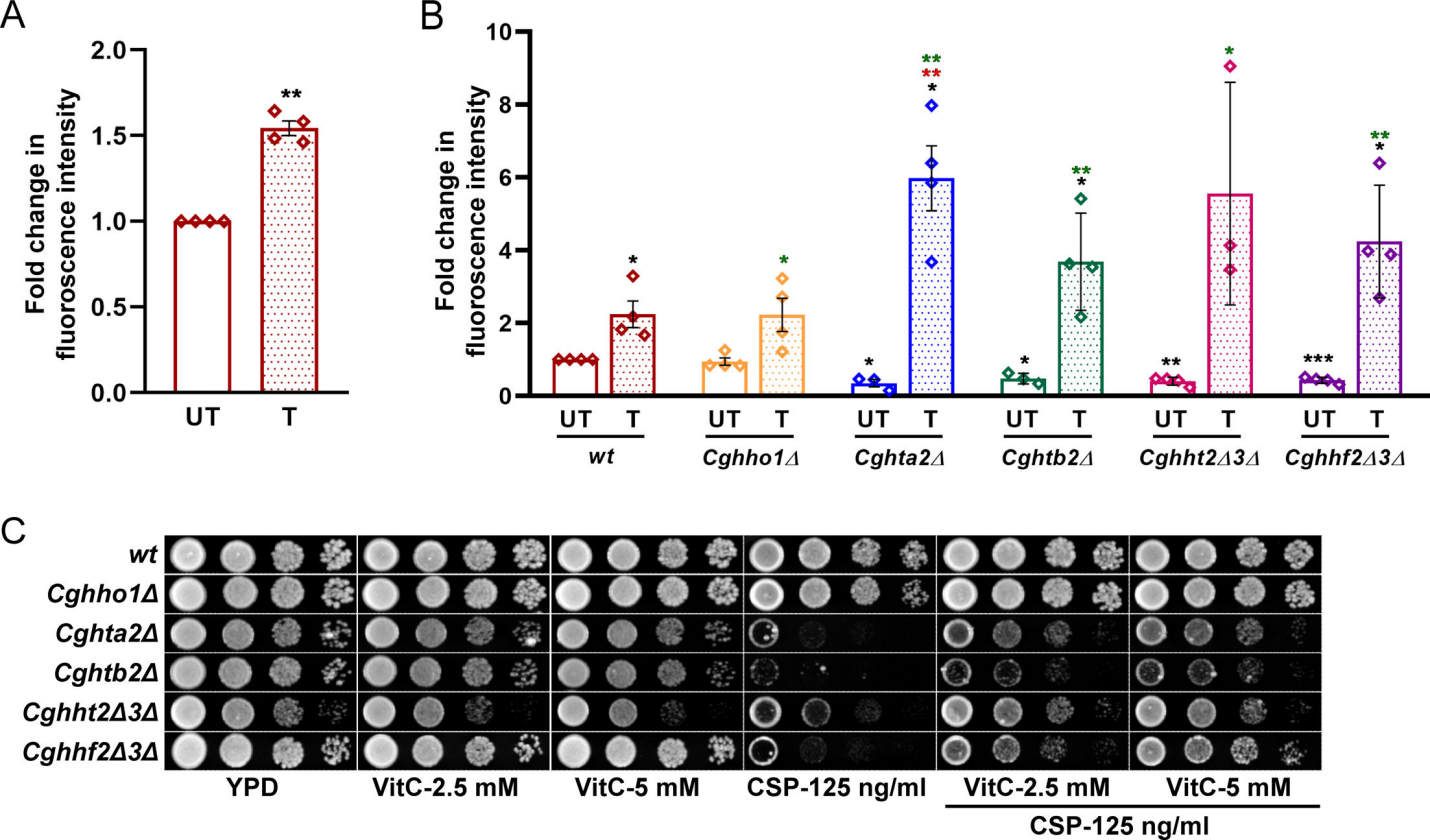
Caspofungin treatment increases cellular ROS levels. (**A**) Intracellular ROS production in caspofungin-treated *wt* cells. Log-phase YPD-grown *wt* cells were either treated (**T**) with caspofungin (200 ng/mL) for 1 h or left untreated (UT), followed by cell incubation with 2′,7′ dichlorodihydrofluorescein diacetate (DCFH-DA) for 30 min at 30°C and 400 rpm. Post-incubation, the cells were washed thrice with PBS, and fluorescence intensity was measured at excitation of 488 nm and emission of 530 nm. Data (mean ± SEM; *n* = 4) represent fluorescence intensities of caspofungin-treated *wt* cells, compared with untreated cells (taken as 100). ** *P* ≤ 0.01; paired two-tailed Student’s *T* test. (**B**) ROS levels in caspofungin-treated indicated *C. glabrata* strains. Fluorescence intensities of caspofungin-treated cells were normalized with untreated *wt* cells (taken as 1.0). Data (mean ± SEM; *n* = 3–4) represent fold change in fluorescence intensity values. Black, red, and green asterisks denote ROS level differences between untreated *wt* and untreated mutant strains, between caspofungin-treated *wt* and caspofungin-treated mutants, and between untreated mutant and caspofungin-treated mutant strains, respectively. *P ≤ 0.05; **P ≤ 0.01; ***P ≤ 0.005, paired and unpaired two-tailed Student’s t test. (**C**) Serial dilution spot assay illustrating the growth of *Cghho1Δ*, *Cghta2Δ*, *Cghtb2Δ*, *Cghht2Δ3Δ,* and *Cghhf2Δ3Δ* mutants in the indicated media. VitC, Vitamin C.

In light of these findings, we next examined the alternate modes by which caspofungin could affect cell growth. Caspofungin has been reported to produce reactive oxygen species (ROS) in *C. glabrata* in a 1,3-β-D-glucan synthase (target enzyme of caspofungin drug)-dependent manner (22). Thus, we first checked if caspofungin exposure leads to ROS production. We found a 1.5-fold to 2-fold increase in ROS levels in caspofungin-treated *wt* cells, compared with untreated *wt* cells (Fig. 7A and B). Next, we checked ROS levels in histone mutants, *Cghho1Δ*, *Cghta2Δ*, *Cghtb2Δ*, *Cghht2Δ3Δ,* and *Cghhf2Δ3Δ*. We observed that although the basal ROS levels were similar in *wt* and *Cghho1Δ* mutant, these were about 2-fold to 3-fold lower in *Cghta2Δ*, *Cghtb2Δ*, *Cghht2Δ3Δ,* and *Cghhf2Δ3Δ* mutants, compared with the *wt* strain (Fig. 7B). Furthermore, caspofungin treatment resulted in a 2-fold, 2-fold, 17-fold, 8-fold, 14-fold, and 10-fold increase in ROS levels in *wt*, *Cghho1Δ*, *Cghta2Δ*, *Cghtb2Δ*, *Cghht2Δ3Δ,* and *Cghhf2Δ3Δ* strains, respectively (Fig. 7B). Importantly, the fold increase in ROS levels in caspofungin-treated *wt* and *Cghho1Δ* mutant, which did not exhibit increased caspofungin susceptibility (Fig. 3), was similar (Fig. 7B). Contrarily, the caspofungin-treated *Cghta2Δ*, *Cghtb2Δ*, *Cghht2Δ3Δ,* and *Cghhf2Δ3Δ* cells contained ~2-fold to 3-fold higher ROS levels, compared with caspofungin-treated *wt* cells (Fig. 7B). These results suggest that the inability of the core histone mutants to grow in the presence of caspofungin could be due to increased ROS levels.

To corroborate the above results, we next checked if the addition of the anti-oxidant, Vitamin C, could restore the attenuated growth of *Cghta2Δ*, *Cghtb2Δ*, *Cghht2Δ3Δ,* and *Cghhf2Δ3Δ* mutants in the medium containing caspofungin. We found that *Cghta2Δ*, *Cghtb2Δ*, *Cghht2Δ3Δ,* and *Cghhf2Δ3Δ* mutants grew better when the medium contained caspofungin along with Vitamin C (Fig. 7C). However, the growth defect reversal was not complete in the Vitamin C-supplemented medium containing caspofungin (Fig. 7C). As a control, we also checked the growth of other core histone mutants, *Cghta1Δ*, *Cghtb1Δ*, *Cghht1Δ2Δ,* and *Cghhf1Δ2Δ*, upon supplementation of Vitamin C. We found similar results, with Vitamin C conferring a growth advantage to the mutants, *albeit* to different extents (Fig.S5). These results suggest that the caspofungin susceptibility of the core histone mutants is in part due to increased cellular ROS levels, which could be due to elevated ROS production and/or impaired ROS detoxification.

### Histone H3K56 acetyl transferase is required for caspofungin tolerance

**Fig 8:**
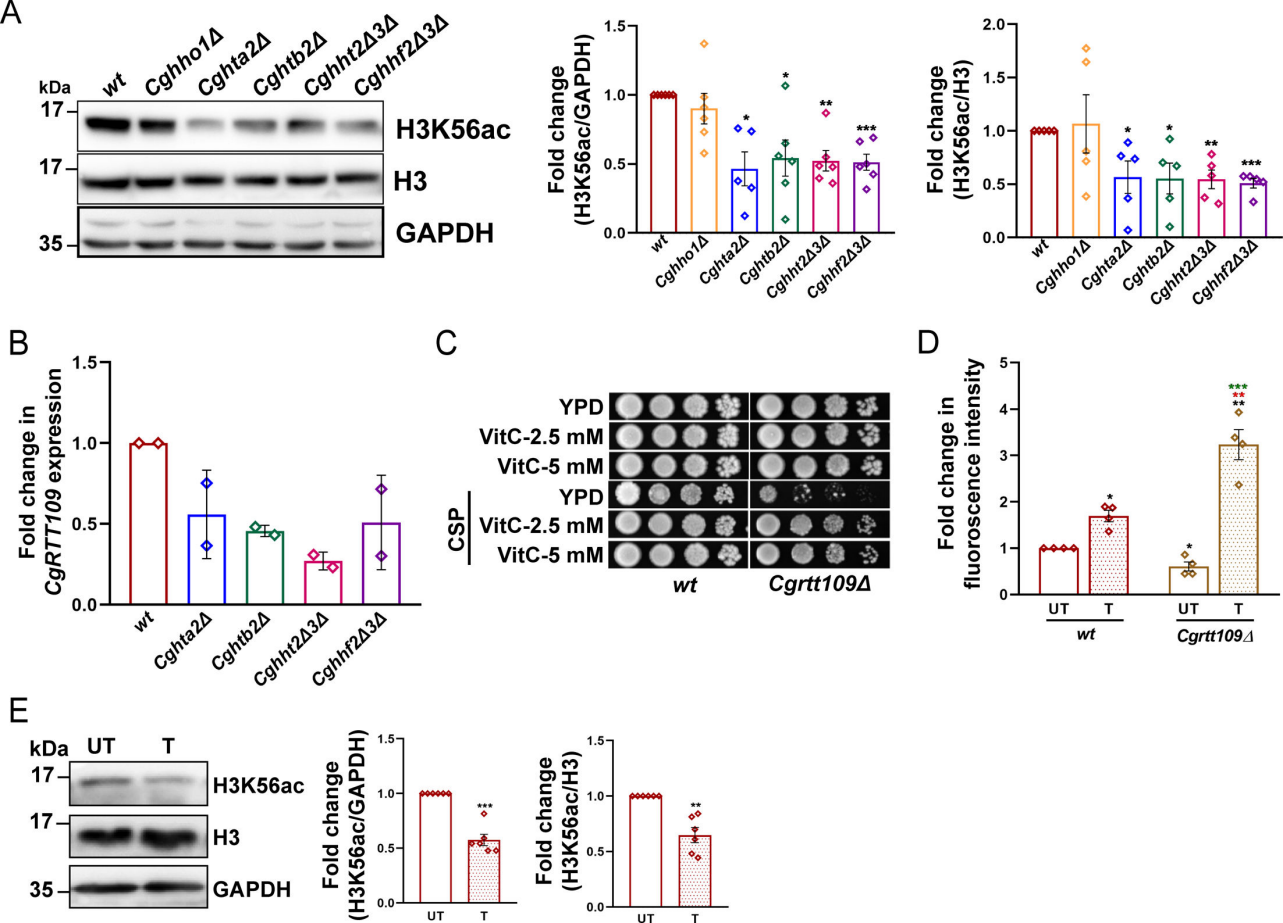
Caspofungin treatment decreases histone H3 acetylation at lysine-56 residue (H3K56ac). (**A**) Representative western blots showing H3K56 acetylation levels in indicated *C. glabrata* strains. Whole cell lysates (60 µg) were resolved on 15% SDS-PAGE and probed with indicated antibodies. For quantification, signal intensity in each lane was measured using ImageJ software, and H3K56ac signal levels were normalized against CgGapdh (used as loading control) and histone H3 signals for each strain. Data (mean ± SEM; *n* = 5) represent fold change in H3K56ac levels in histone mutants, compared with *wt* (taken as 1.0), and are plotted on the right side of the blot images. * *P* ≤ 0.05; **P ≤ 0.01; ***P ≤ 0.005; paired two-tailed Student’s *t* test. (**B**) qRT-PCR-based analysis of *CgRTT109* transcript levels in log-phase cells of indicated strains, as determined by 2^-ΔΔ^C_T_ method, using *CgACT1* mRNA as control. Data (mean ± SD; *n* = 2) represent fold change in *CgRTT109* transcript levels in histone mutants, compared with *wt* cells (taken as 1.0). (**C**) Serial dilution spot assay illustrating the growth of the *Cgrtt109Δ* mutant in the indicated medium. Caspofungin (CSP) was added to a final concentration of 150 ng/mL. VitC, Vitamin C. (**D**) DCFH-DA-based measurement of ROS levels in log-phase *Cgrtt109Δ* cells. Data (mean ± SEM; *n* = 3–4) represent the fluorescence intensity of *Cgrtt109Δ* cells, compared with *wt* cells (taken as 1.0). Black, red, and green asterisks denote ROS level differences between untreated *wt* and untreated *Cgrtt109Δ* cells, between caspofungin-treated *wt* and caspofungin-treated *Cgrtt109Δ*, and between untreated *Cgrtt109Δ* and caspofungin-treated *Cgrtt109Δ*, respectively. *P ≤ 0.05; **P ≤ 0.01; ***, *P* ≤ 0.005; paired and unpaired two-tailed Student’s *t* test. (**E**) Representative western blots showing H3K56 acetylation levels in caspofungin-treated *wt* cells. Log-phase *wt* cells were treated with 200 ng/mL caspofungin (**T**) for 1 h or left untreated (UT). Whole cell lysates (60 µg) were resolved on 15% SDS-PAGE and probed with indicated antibodies. For quantification, signal intensity in each lane was measured using ImageJ software, and H3K56ac signal levels were normalized against CgGapdh (used as loading control) and histone H3 signals for both conditions. Data (mean ± SEM; *n* = 6) represent fold change in H3K56ac levels in caspofungin-treated *wt* cells, compared with untreated *wt* cells (taken as 1.0), and are plotted on the right side of the blot images. ** *P* ≤ 0.01; ***P ≤ 0.005; paired two-tailed Student’s *t* test.

During replication, the newly synthesized histone H3 in *S. cerevisiae* is acetylated at lysine-56 residue by Rtt109 (H3K56 acetyl transferase), in conjunction with the nucleosome assembly factor Asf1, with both Rtt109 and Asf1 being pivotal to gene expression homeostasis (28, 29). In *C. glabrata*, CgRtt109 has been shown to be required for replication in macrophages, survival of MMS and oxidative stress, and acetylation of histone H3 at lysine-56 residue (30). Since histone H3 lysine 56 acetylation (H3K56Ac) loss has previously been linked with decreased echinocandin tolerance in *C. albicans* (31), we sought to determine if H3K56Ac levels were altered in the core histone mutants. For this, we performed western blot analysis using anti-H3K56Ac antibody. We found a ~50% reduction in basal H3K56Ac levels in caspofungin-sensitive *Cghta2Δ*, *Cghtb2Δ*, *Cghht2Δ3Δ,* and *Cghhf2Δ3Δ* mutants, compared with *wt* cells (Fig. 8A). Notably, the *Cghho1Δ* mutant, which showed *wt*-like caspofungin susceptibility, had H3K56Ac levels similar to those in *wt* cells (Fig. 8A). Consistent with a diminished acetylation of histone H3 at the lysine-56^th^ residue, we found that *CgRTT109* transcript levels were 40%–70% lower in *Cghta2Δ*, *Cghtb2Δ*, *Cghht2Δ3Δ,* and *Cghhf2Δ3Δ* mutants, compared with the *wt* strain (Fig. 8B). These data suggest that caspofungin susceptibility of the core histone mutants could in part be due to reduced *CgRTT109* levels.

To corroborate these findings further, we performed three experiments. First, we checked caspofungin susceptibility of the *Cgrtt109Δ* mutant, which lacks histone H3K56 acetyltransferase (30). We found that the *Cgrtt109Δ* mutant could not grow in the presence of caspofungin, and this growth defect was rescued when caspofungin-containing medium was supplemented with Vitamin C (Fig. 8C). Second, the *Cgrtt109Δ* mutant contained 2-fold lower basal ROS levels; however, the caspofungin-treated *Cgrtt109Δ* cells displayed 1.5-fold higher ROS levels, compared with the caspofungin-treated *wt* cells (Fig. 8D). Of note, these phenotypes of the *Cgrtt109Δ* mutant are similar to those exhibited by the core histone mutants, which showed increased susceptibility to caspofungin. Finally, caspofungin exposure led to a 35% reduction in H3K56Ac levels in *wt* cells (Fig. 8E). These results underscore that H3K56Ac modification governs caspofungin susceptibility in *C. glabrata*.

**Fig 9:**
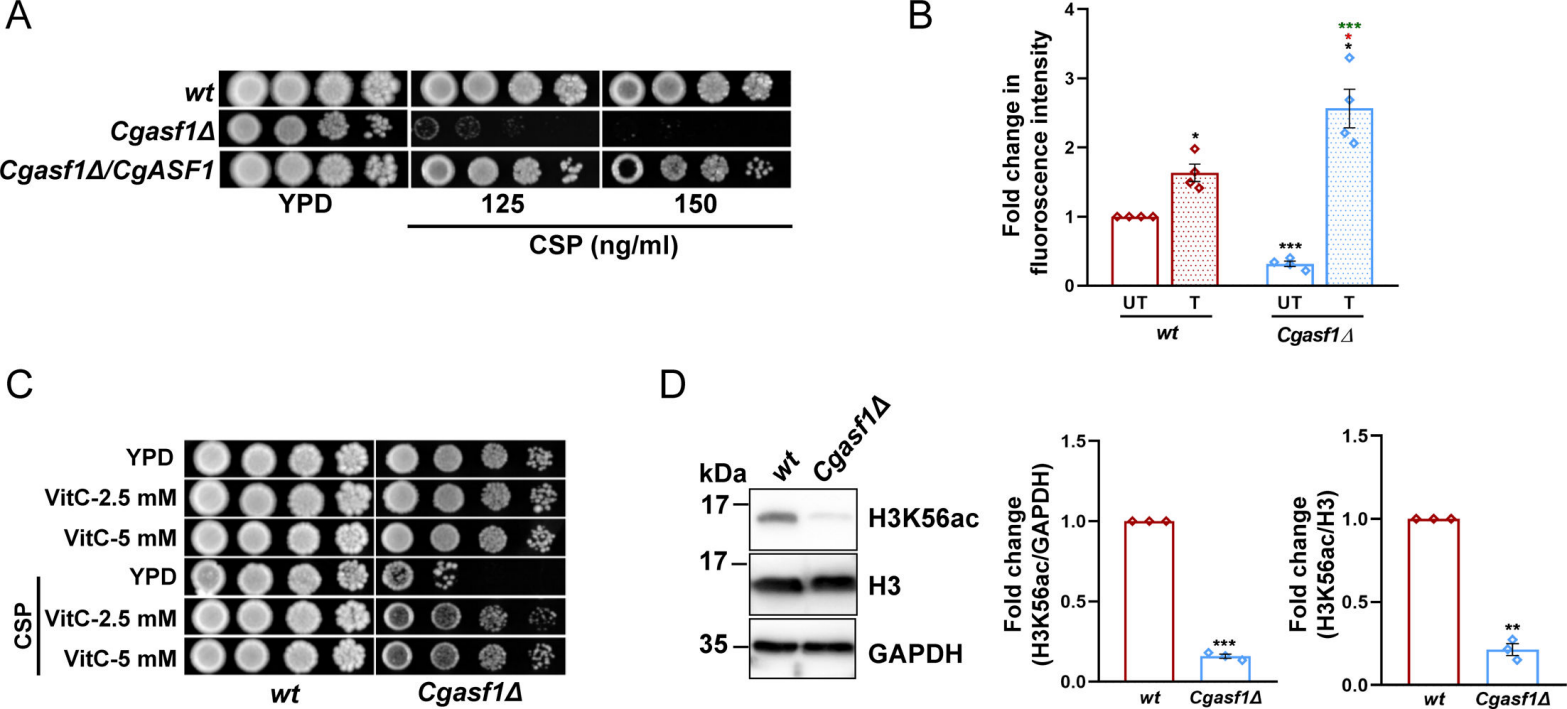
The *Cgasf1Δ* mutant displays increased caspofungin susceptibility and diminished H3K56 acetylation (**A**) Serial dilution spot assay illustrating attenuated growth of the *Cgasf1Δ* mutant in the medium containing caspofungin. (**B**) DCFH-DA-based measurement of ROS levels in log-phase *Cgasf1Δ* cells. Data (mean ± SEM; *n* = 4) represent the fluorescence intensity of *Cgasf1Δ* cells, compared with *wt* cells (taken as 1.0). Black, red, and green asterisks denote ROS level differences between untreated *wt* and untreated *Cgasf1Δ*, between caspofungin-treated *wt* and caspofungin-treated *Cgasf1Δ*, and between untreated *Cgasf1Δ* and caspofungin-treated *Cgasf1Δ*, respectively. *P ≤ 0.05; ***, *P* ≤ 0.005; paired and unpaired two-tailed Student’s t test. (**C**) Serial dilution spot assay illustrating growth of the *Cgasf1Δ* mutant in the vitamin C-supplemented medium. Caspofungin (CSP) was added to a final concentration of 150 ng/mL. VitC, Vitamin C. (**D**) Representative western blots showing diminished H3K56 acetylation levels in *Cgasf1Δ* mutant. Whole cell lysates (60 µg) were resolved on 15% SDS-PAGE and probed with indicated antibodies. For quantification, signal intensity in each lane was measured using ImageJ software, and H3K56ac signal levels were normalized against CgGapdh (used as loading control) and histone H3 signals for each strain. Data (mean ± SEM; *n* = 3) represent fold change in H3K56ac levels in *Cgasf1Δ* cells, compared with *wt* cells (taken as 1.0), and are plotted on the right side of the blot images. ** *P* ≤ 0.01; ***, *P* ≤ 0.005; paired two-tailed Student’s *t* test.

To further strengthen this notion, we generated *Cgasf1Δ* mutant, which lacked the nucleosome assembly factor CgAsf1, and checked its susceptibility to caspofungin. In *S. cerevisiae*, the histone chaperone Asf1 and the histone H3K56 acetyltransferase Rtt109 work in concert to maintain the chromatin structure, with Asf1 stimulating the H3 acetyl transferase activity of Rtt109 (11, 28, 29). Asf1 in *S. cerevisiae* is also pivotal to repression of the histone gene transcription during the cell cycle (32). However, Asf1 functions in *C. glabrata* are not well understood. Our phenotypic analysis of the *Cgasf1Δ* mutant revealed that the *Cgasf1Δ* mutant was unable to grow well in the presence of caspofungin (Fig. 9A), contained low basal ROS levels (Fig. 9B), and exhibited elevated ROS levels upon caspofungin exposure (Fig. 9B). Importantly, Vitamin C supplementation rescued the increased caspofungin susceptibility of the *Cgasf1Δ* mutant (Fig. 9C). Furthermore, western blot analysis revealed that H3K56Ac is highly reduced in the *Cgasf1Δ* mutant, compared with *wt* cells (Fig. 9D). These phenotypes of the *Cgasf1Δ* mutant mirror the effects of the reduced core histone gene dosage as well as of the CgRtt109 enzyme loss, thereby implicating H3K56Ac in regulating caspofungin susceptibility in *C. glabrata*.

**Fig 10:**
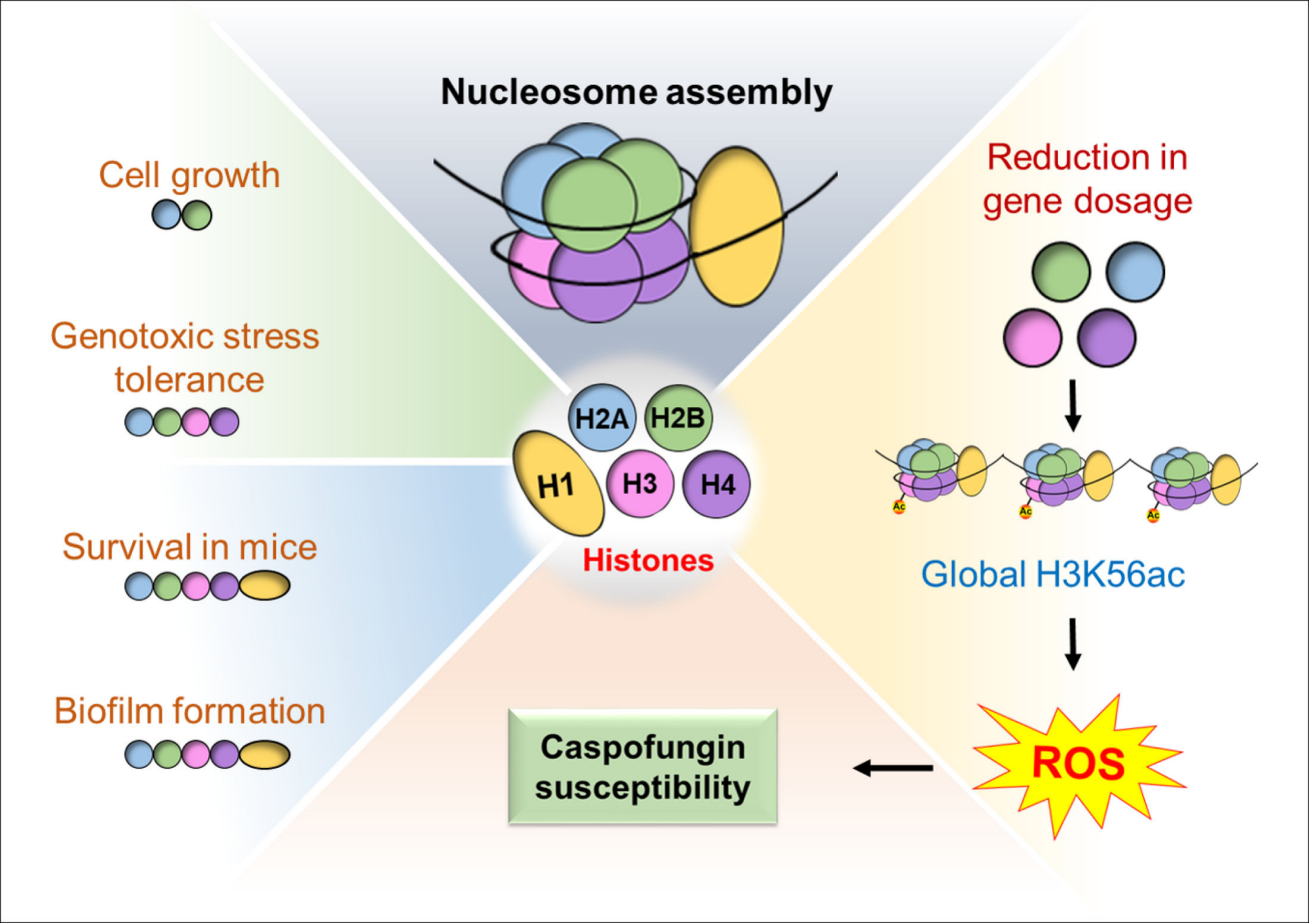
A schematic illustrating major findings of the study. A reduction in core histone gene dosage leads to diminished H3K56Ac as well as increased ROS production upon caspofungin treatment, which leads to increased caspofungin susceptibility. Additionally, lower levels of histone proteins differentially impact cellular processes, namely, cell proliferation, biofilm formation, MMS stress survival, and survival in the murine model of systemic candidiasis.

Although it remains to be demonstrated how a reduction in core histone levels governs *CgRTT109* levels, it is possible that a perturbed stoichiometry of histone proteins adversely affects the nucleosome assembly and/or positioning, which may downregulate *CgRTT109* transcription. Altogether, our findings show that the echinocandin stress leads to a reduction in histone H2A and H2B levels as well as in H3K56Ac levels. The involvement of H3K56 acetylation in cellular adaptability and response to the caspofungin drug highlights that the low histone levels may indirectly alter histone modifications, thereby affecting the transcriptional factor recruitment and activation/repression of caspofungin-responsive genes. Furthermore, the imbalanced histone supply may perturb the nucleosome assembly, thereby influencing multiple processes, including ROS detoxification, and rendering *C. glabrata* cells susceptible to caspofungin (Fig. 10). Finally, our data show that the core histone protein levels regulate the ability of *C. glabrata* cells to form biofilms, survive in the mouse model of systemic candidiasis, and thrive under DNA damage stress *in vitro* (Fig. 10), thereby uncovering histone-regulated attributes that may promote drug resistance and pathogenesis of *C. glabrata*. Collectively, our findings add to the growing body of evidence of a regulatory role for histone homeostasis in governing the expression of the genes that promote tolerance/resistance to antifungal drugs. Since histone H3K56 acetylation is pivotal to the cellular antioxidant defense system, fungal-specific inhibitors of the Rtt109 enzyme hold promise as new antifungal agents.

## DISCUSSION

Fungal infections do not garner enough attention, despite 2.5 million people dying of invasive mycoses every year (33). The limited antifungal arsenal and the ever-increasing incidence of drug-resistant fungal isolates are emerging as significant issues in the field of medical mycology. Herein, we dissect the link between histone gene dosage and fungal response to the cell wall-targeting caspofungin drug and report for the first time that the core histone gene dosage modulates caspofungin tolerance, with lower histone protein levels rendering *C. glabrata* cells more susceptible to caspofungin.

Histones, core components of the chromatin, influence cellular stress responses by regulating gene expression, protecting DNA from damage and promoting DNA repair, thereby facilitating adaptation and survival processes to varied stresses (2, 19, 23). Since changes in histone gene dosages are associated with altered chromatin structure and functions, histone levels are tightly regulated to maintain optimal chromatin dynamics. Earlier studies have shown that a reduction in histone gene dosage in *S. cerevisiae* impacts genotoxic stress susceptibility and genome stability (34, 35). Consistent with this, the substantially reduced histone H4 levels led to highly attenuated growth and resistance to DNA damage stress in *C. albicans* and *C. glabrata*, respectively (7, 8). Contrarily, a low histone H3 gene dosage in *C. glabrata* resulted in DNA damage sensitivity (8). Recently, a reduced dosage of histone H3 and H4 in *C. albicans* was found to be associated with calcofluor white sensitivity, elevated ROS levels, and diminished renal fungal burden in the murine systemic candidiasis model (9). Altogether, these findings underscore the variable effects of altered levels of core histones on cellular stress responses. Our data add another layer to this histone dosage cellular network by demonstrating that while a complete loss of the linker histone CgHho1 had no effect on cell physiology of *C. glabrata*, a reduction in the core histone proteins, H2A, H2B, H3, and H4, led to diminished ROS levels and elevated caspofungin sensitivity (Fig. 3 and 7B).

Furthermore, histone post-translational modifications regulate the expression of the core histone genes (36), with the growing evidence underscoring the important role of epigenetic changes in fungal adaptation to the drug stress (19, 23). For example, the *C. albicans hat1Δ/Δ* mutant, lacking the histone H4 acetyl transferase Hat1, displayed exquisite susceptibility to caspofungin due to excessive ROS production (37). Similarly, Rtt109, which acetylates histone H3 on K56, has been implicated in oxidative stress response and 5-fluorocytosine and echinocandin susceptibility in *C. albicans* (38). Moreover, the lack of Snf2, the SWI/SNF complex catalytic subunit, resulted in azole sensitivity in *C. albicans* and *C. glabrata* (24, 39). Similarly, Gcn5, a catalytic subunit of the ADA and SAGA histone acetyltransferase complexes, modulated the tolerance to echinocandins and azole and echinocandin drugs in *C. albicans* and *C. glabrata*, respectively (40, 41). All these findings have led to the exploration of the inhibitors of the histone deacetylases as potential adjunctive therapies for drug-resistant fungal isolates (19).

Notably, two recent studies in *C. glabrata* have also highlighted the role of histone methylation modifications in regulating antifungal susceptibility, with histone H3K4 methyltransferase (CgSet1) and H3K36 methyltransferase (CgSet2) loss leading to increased and decreased azole susceptibility, respectively (21, 42). Intriguingly, although fluconazole treatment in *C. glabrata* led to an increase in histone H3 and H4 protein levels (43), caspofungin exposure resulted in decreased expression of histone H2A and H2B in the current study (Fig. 5). Of note, both histone H3 and H4 transcript levels have been reported to be decreased and increased, respectively, in response to replication stress and lower temperature (25^ο^C) in *S. cerevisiae* and *Cryptococcus neoformans* (35, 44). Although these histone level changes likely reflect the cellular response to a specific stress, the impact of these alterations on nucleosome assembly, chromatin functions, and gene expression must be determined.

Caspofungin treatment is known to lead to cell wall stress and ROS production (19, 22). The protein kinase C-mediated cell wall integrity pathway has been reported to intersect with ROS signaling (41). Recovery/adaptation to caspofungin-imposed cell wall stress possibly involves eliminating damaged cell wall constituents, elevated synthesis, and incorporation of the major structural cell wall component β-glucan, and an increase in the cell wall chitin (19, 23). Although *C. glabrata* mutants with reduced core histones exhibited no gross cell wall defect, as reflected in their *wt*-like sensitivity to zymolyase digestion (Fig. S4), they contained lower ROS levels (Fig. 7B). Furthermore, the antioxidant Vitamin C-mediated rescue of the caspofungin susceptibility of the core histone mutants (Fig. 7C), *Cgrtt109Δ* mutant (Fig. 8C), and *Cgasf1Δ* mutant (Fig. 9C) suggests that their susceptibility is likely to be due to excessive ROS production and/or inadequate antioxidant defense systems. Thus, maintaining the balance between ROS generation and scavenging appears to be of paramount importance to ensure cell survival upon caspofungin stress. In this regard, it is also possible that owing to histones’ role in regulating DNA accessibility for DNA repair, recombination, and gene activation processes, these mutants with an aberrant chromatin architecture and a perturbed nucleosome assembly are unable to generate the requisite transcriptional landscape to counteract caspofungin stress. Alternately, a reduction in H3K56Ac (an active chromatin mark) levels leads to an inadequate transcriptional response to caspofungin stress. Thus, future studies will investigate how histone protein shortage and/or diminished H3K56Ac results in perturbed nucleosome assembly, aberrant global gene expression, and an impaired cellular ability to repair oxidative stress-induced damage.

Finally, the linker histones (Hho1) bind to the linker DNA, contribute to the higher-order chromatin structure, and perform both architectural and regulatory functions (3). However, *CgHHO1* loss resulted in no discernible effect on cell growth and antifungal susceptibility in *C. glabrata* (Fig. 1A and 3). In this regard, it is worth noting that a recent single-molecule force spectroscopy study in *S. cerevisiae* showed that the HMG-box protein Hmo1, instead of Hho1, promotes the nucleosome assembly on DNA in yeast nucleoplasmic extracts (45). Thus, it is possible that CAGL0E00737p, the *C. glabrata* ortholog of *S. cerevisiae* Hmo1, can adequately perform chromatin assembly functions *in vivo* in the absence of CgHho1, thereby maintaining cellular homeostasis under regular and stressful conditions. Alternately, the possible relaxed chromatin architecture, devoid of higher-order structures, in the *Cghho1Δ* mutant, as reported for the *S. cerevisiae hho1Δ* mutant (46), is capable of responding to caspofungin-induced stress via regulated transcriptional activity.

Altogether, our findings uncover an intricate relationship between the core histone gene dosage and the histone H3K56 acetylation and show how this interplay governs ROS detoxification and cell wall dynamics upon caspofungin treatment. Our findings also suggest that the compounds impeding histone synthesis and/or perturbing histone post-translational modifications hold potential as promising antifungal adjuvants.

## MATERIALS AND METHODS

### Strains and culture conditions

*C. glabrata* strains were grown in Yeast Peptone Dextrose (YPD) medium, which is a rich medium for yeast cell growth. These strains were grown at 30°C either under static or shaking (200 rpm) conditions. Complemented strains, which carried plasmids with the *URA3* gene as a selection marker, were grown in a CAA (Cas Amino Acid) medium, which lacked uracil.

*Escherichia coli* DH5α strains were grown in the LB medium supplemented with appropriate antibiotics for both liquid and solid media. Bacterial strains, grown either under static or shaking conditions at 37°C, were used for gene cloning and plasmid propagation studies.

Logarithmic-phase (log-phase) *C. glabrata* cells were collected by culturing overnight-grown *C. glabrata* cells in the fresh appropriate medium (YPD/CAA) for 4–5 h at 30°C under shaking conditions. For this, overnight cultures were inoculated at an initial OD_600_ of 0.1.

### *C. glabrata* strain construction

*C. glabrata* deletion mutants were generated by replacing the gene of interest with the *nat1* gene using a homologous recombination-based approach, and nourseothricin resistance was used to select transformants, as described previously (8). For complementation analysis, *C. glabrata* mutant strains were transformed with pCU-PDC1 (pRK999) plasmid carrying the gene of interest. Histone H4 mutant complementation analysis was carried out by expressing the *CgHHF* gene from the native promoter, as described previously (8). All transformants were selected for uracil prototrophy. Multiple transformants were tested for complementation studies. *C. glabrata* strains, plasmids, recombinant vectors, oligonucleotides, antibodies and chemicals, commercial kits, and software used in this study are listed in Tables S2 to S5.

### THP-1 cells

THP-1 is a human monocytic cell line that is derived from a 1-year-old acute monocytic leukemia patient. THP-1 monocyte cells (TIB-202) were obtained from ATCC and differentiated into macrophages upon PMA (phorbol-12-myristate-13-acetate; 16 nM) treatment. THP-1 cells were cultured in the complete RPMI-1640 medium containing 10% heat-denatured FBS (fetal bovine serum), 2 mM glutamine, 100 units/mL penicillin, 100 µg/mL streptomycin at 37°C, and 5% CO_2_ in a tissue culture incubator.

### Serial dilution spot assay

*C. glabrata* strains were grown in YPD/CAA broth and incubated at 30°C with shaking. After measuring OD_600_, overnight cultures were suspended in PBS (phosphate-buffered saline), and OD_600_ was normalized to 1.0. Cell suspensions were diluted four times by a 10-fold serial dilution in PBS; 3 µL of each diluted culture was spotted on an appropriate growth medium lacking or containing a stress-causing agent. Plates were incubated at 30 ^ο^C in static condition during the analysis period, and images were captured every 24 h. Heat map was generated by using the matrix2png web tool (https://matrix2png.msl.ubc.ca/). Fitness score was assigned to each mutant based on the comparative growth analysis of the mutant and the *wt* strain under the same condition.

### Growth curve analysis

*C. glabrata* strains were grown overnight in YPD broth and inoculated in fresh YPD medium at an initial OD_600_ of 0.1. Cultures were incubated at 30°C under shaking conditions. Culture absorbance was recorded at regular intervals till 36 h. OD_600_ values of each strain were plotted against time. Generation time was calculated during the log phase (2–8 h) of the growth.

For dose-response curve analysis, the filter-sterilized, RPMI 1640 medium lacking sodium bicarbonate and containing 2% glucose and 0.165 M MOPS [3-(N-morpholino) propanesulfonic acid] was used to grow *C. glabrata* cells as well as to prepare caspofungin drug solution. Cells were grown in different caspofungin concentrations, viz., 1, 2, 4, 8, 16, 32, 64, 128, 256, 512, and 1024 ng/mL for 24 h at 37°C, and the culture absorbance was recorded at 530 nm.

### Biofilm formation

*C. glabrata* strains were grown overnight in YPD medium at 30 ^ο^C in shaking condition. Cells were collected and suspended in a complete RPMI-1640 medium. 0.5 OD_600_ cells were added to a well of a 24-well polystyrene plate and incubated at 37°C for 24 h. After 24 h of incubation, 500 µL of the spent medium was removed slowly, without disturbing *C. glabrata* cells at the bottom of the well; 500 µL fresh RPMI-1640 medium was added to each well, and cells were incubated at 37 ^ο^C for another 24 h. Next, the culture supernatant was carefully aspirated, without disturbing the biofilm formed at the bottom of the well. The wells containing *C. glabrata* biofilms were gently washed twice with PBS. After drying the plate completely at 37°C, the biofilm was stained with 500 µL of 0.4% crystal violet (wt/vol; in 20% ethanol) at room temperature for 45 min. Post-incubation, the crystal violet stain was removed by pipetting off the liquid, and the wells were washed thrice with water. Biofilm was de-stained by adding 95% ethanol and incubating for 45 min at room temperature. The biofilm mass in each well was determined by measuring the absorbance of the destained solution at 595 nm and normalizing the value obtained by subtracting the absorbance of the blank well containing no cells. Biofilm formation was plotted as the ratio of the absorbance of the mutant strain to that of the *wt* (wild-type) strain. A difference of at least 20% in biofilm formation between *wt* and mutants was considered significant.

### Macrophage infection assay

PMA-treated THP-1 cells (1 × 10^6^ cells) were seeded in a well of a 24-well plate. After 12 h of PMA treatment, followed by 12 h of recovery, THP-1 cells were infected with *C. glabrata* strains at a multiplicity of infection (MOI) of 10:1 and incubated at 37°C, 5% CO_2_ in a tissue culture incubator; 2 h post-infection, THP-1 cells were washed thrice with PBS to remove unphagocytosed *C. glabrata* cells, and the fresh pre-warmed RPMI-1640 medium was added. At 2 h and 24 h post-infection, cells were washed thrice with PBS, and 1 mL sterile water was added for osmolysis of THP-1 cells, followed by well scraping and collecting macrophage lysates containing *C. glabrata* cells. THP-1 cell lysates were diluted appropriately and plated on YPD medium. Plates were incubated at 30°C for 48 h. The colonies were counted, and the final CFUs (colony-forming units) were calculated by multiplying the colony number by the appropriate dilution factor. Fold replication was calculated by dividing CFUs at 24 h with CFUs at 2 h, whereas the phagocytosis rate was calculated as CFUs at 2 h divided by CFUs at 0 h. A < 25% difference in fold replication between *wt* and mutants was not considered significant.

### Mice infection assay

Overnight-grown *C. glabrata* strains were washed twice with sterile PBS, and the cell density was adjusted to 20 OD_600_; 100 µL cell suspension was injected into 6- 8-week-old female BALB/c mice through the tail vein. On the seventh day post-infection, mice were sacrificed, and kidneys, liver, and spleen were excised for fungal burden examination. Organs were homogenized in PBS, and appropriate dilutions were plated on YPD medium containing antibiotics (penicillin and streptomycin). Plates were incubated at 30°C for 1–2 days. Colonies were counted, and the CFUs were calculated for each *C. glabrata* strain by multiplying the colony number by the respective dilution factor. Mice were infected and observed at the BRIC-CDFD Experimental Animal Facility, Hyderabad, India. All guidelines of the Committee for the Purpose of Control and Supervision of Experiments on Animals, Government of India, were strictly followed. The protocol was approved by the CDFD Animal Ethics Committee (EAF/RK/32/2022).

### Immunoblotting analysis

Log-phase *C. glabrata* cells were grown in YPD medium lacking or containing 200 ng/mL caspofungin for 1 h. Whole cell extracts were prepared by glass bead lysis, and proteins were resolved on 12% or 15% SDS-PAGE and probed with appropriate antibodies. CgGapdh was used as a loading control. All band intensities were quantified using ImageJ software.

### Cell wall composition analysis

*C. glabrata* strains were grown overnight in the YPD medium at 30°C. Cultures were re-inoculated in the YPD medium at an initial OD_600_ of 0.1. After 3–4 h of growth at 30°C, log phase cells (2.0 OD_600_) were collected, washed, and suspended in PBS. Cells were stained with calcofluor white (2.5 µg/mL), aniline blue (50 mg/mL), or FITC-conjugated concanavalin A (1 mg/mL) for 30 min at room temperature for chitin, glucan, and mannan estimation, respectively. After PBS washes, fluorescence intensity of ~50,000 cells was recorded using the BD LSRFortessa X-20 flow cytometer. Data were analyzed using BD FACSDIVA v9.0 software. The background fluorescence was corrected by subtracting the mean intensity fluorescence values of unstained samples from that of the respective stained samples.

### Intracellular ROS determination

Overnight-grown *C. glabrata* strains were re-inoculated in the YPD medium for 3–4 h and treated either with caspofungin at 200 ng/mL or left untreated for 1 h. The total number of cells in the log-phase cultures of *wt*, *Cghho1Δ*, *Cghta2Δ*, *Cghtb2Δ*, *Cghht2Δ3Δ,* and *Cghhf2Δ3Δ* strains was measured using the hemocytometer. An OD_600_ of 1.0 corresponded to approximately 2 × 10^7^ cells/mL for all strains. For ROS estimation, cells corresponding to 3.0 OD_600_ were collected and washed with PBS, followed by incubation with 10 µM DCFH-DA (2′,7′-Dichlorodihydrofluorescein diacetate) at 30°C for 30 min in the dark. After 30 min, cells were washed thrice with PBS and re-suspended in PBS. The fluorescence intensity values were measured at an excitation and an emission wavelength at 488 nm and 530 nm, respectively (47).

### Zymolyase digestion assay

Log-phase YPD medium-grown *C. glabrata* cells (2.0 OD_600_) were collected and washed with PBS. Cells were treated with 50 µg/mL zymolyase at 30°C in a 96-well plate. The absorbance was recorded at 600 nm at every 10-min interval over a period of 6 h. The absorbance at 0 min was considered 100, and the absorbance at each time point value was plotted as a decrease in absorbance relative to the 0 min value (47).

### Quantitative RT-PCR (qRT-PCR) analysis

Log-phase *C. glabrata* cells were grown in YPD medium. RNA was extracted using the acid-phenol method, followed by DNase I digestion; 500 ng DNase I-treated total RNA was used for cDNA synthesis using the Superscript III reverse transcriptase. qRT-PCR was performed using the DyNAmo ColorFlash SYBR Green qPCR kit (Thermo Scientific), and gene expression was determined by the 2^-ΔΔCT^ method. The housekeeping *CgACT1* gene was used as a control.

### Statistical analysis

The GraphPad Prism software application was used for statistical significance determination using the two-tailed Student *t*-test or the nonparametric Mann-Whitney test. The error bar indicates standard error of the mean (SEM). Asterisks were used to represent *P* values. The *P* value of ≤ 0.05 was considered to be significant; *P ≤ 0.05, **P ≤ 0.01; ***P ≤ 0.005; ****P ≤ 0.001.

## SUPPLEMENTAL MATERIAL

Supplemental material spectrum.02399-24-s0001.docx Fig. S1 to S5; Supplemental legends.

Table S1 spectrum.02399-24-s0002.xlsx Canonical histone-encoding ORFs in *C. glabrata*.

Table S2 spectrum.02399-24-s0003.xlsx Strains used in the study.

Table S3 spectrum.02399-24-s0004.xlsx Plasmids used in the study.

Table S4 spectrum.02399-24-s0005.xlsx Primers used in the study.

Table S5 spectrum.02399-24-s0006.xlsx Antibodies (A), chemicals, commercial kits and software (B) used in the study.

ASM does not own the copyrights to Supplemental Material that may be linked to, or accessed through, an article. The authors have granted ASM a non-exclusive, world-wide license to publish the Supplemental Material files. Please contact the corresponding author directly for reuse.

## References

[1] Luger K, Mäder AW, Richmond RK, Sargent DF, Richmond TJ. 1997. Crystal structure of the nucleosome core particle at 2.8 A resolution. Nature 389:251–260. doi:10.1038/38444 9305837

[2] Zhou K, Gaullier G, Luger K. 2019. Nucleosome structure and dynamics are coming of age. Nat Struct Mol Biol 26:3–13. doi:10.1038/s41594-018-0166-x 30532059 PMC7386248

[3] Fyodorov DV, Zhou B-R, Skoultchi AI, Bai Y. 2018. Emerging roles of linker histones in regulating chromatin structure and function. Nat Rev Mol Cell Biol 19:192–206. doi:10.1038/nrm.2017.94 29018282 PMC5897046

[4] Weiner A, Hsieh T-HS, Appleboim A, Chen HV, Rahat A, Amit I, Rando OJ, Friedman N. 2015. High-resolution chromatin dynamics during a yeast stress response. Mol Cell 58:371–386. doi:10.1016/j.molcel.2015.02.002 25801168 PMC4405355

[5] Mojica EA, Kültz D. 2022. Physiological mechanisms of stress-induced evolution. J Exp Biol 225:jeb243264. doi:10.1242/jeb.243264 35258607

[6] Cui X, Dard A, Reichheld J-P, Zhou D-X. 2023. Multifaceted functions of histone deacetylases in stress response. Trends Plant Sci 28:1245–1256. doi:10.1016/j.tplants.2023.06.006 37394308

[7] Zacchi LF, Selmecki AM, Berman J, Davis DA. 2010. Low dosage of histone H4 leads to growth defects and morphological changes in Candida albicans. PLoS One 5:e10629. doi:10.1371/journal.pone.0010629 20498713 PMC2869362

[8] Kumar K, Moirangthem R, Kaur R. 2020. Histone H4 dosage modulates DNA damage response in the pathogenic yeast Candida glabrata via homologous recombination pathway. PLoS Genet 16:e1008620. doi:10.1371/journal.pgen.1008620 32134928 PMC7058290

[9] Dong Y, Du J, Deng Y, Cheng M, Shi Z, Zhu H, Sun H, Yu Q, Li M. 2024. Reduction of histone proteins dosages increases CFW sensitivity and attenuates virulence of Candida albicans. Microbiol Res 279:127552. doi:10.1016/j.micres.2023.127552 38000336

[10] Rasheed M, Battu A, Kaur R. 2020. Host-pathogen interaction in Candida glabrata infection: current knowledge and implications for antifungal therapy. Expert Rev Anti Infect Ther 18:1093–1103. doi:10.1080/14787210.2020.1792773 32668993

[11] Fidel PL, Vazquez JA, Sobel JD. 1999. Candida glabrata: review of epidemiology, pathogenesis, and clinical disease with comparison to C. albicans. Clin Microbiol Rev 12:80–96. doi:10.1128/CMR.12.1.80 9880475 PMC88907

[12] Lamoth F, Lockhart SR, Berkow EL, Calandra T. 2018. Changes in the epidemiological landscape of invasive candidiasis. J Antimicrob Chemother 73:i4–i13. doi:10.1093/jac/dkx444 29304207 PMC11931512

[13] Pfaller MA, Diekema DJ, Turnidge JD, Castanheira M, Jones RN. 2019. Twenty years of the SENTRY antifungal surveillance program: results for Candida species from 1997-2016. Open Forum Infect Dis 6:S79–S94. doi:10.1093/ofid/ofy358 30895218 PMC6419901

[14] Meyahnwi D, Siraw BB, Reingold A. 2022. Epidemiologic features, clinical characteristics, and predictors of mortality in patients with candidemia in Alameda County, California; a 2017-2020 retrospective analysis. BMC Infect Dis 22:843. doi:10.1186/s12879-022-07848-8 36371155 PMC9652840

[15] Healey KR, Perlin DS. 2018. Fungal resistance to echinocandins and the MDR phenomenon in Candida glabrata. JoF 4:105. doi:10.3390/jof4030105 30200517 PMC6162769

[16] Arendrup MC, Patterson TF. 2017. Multidrug-resistant Candida: epidemiology, molecular mechanisms, and treatment. J Infect Dis 216:S445–S451. doi:10.1093/infdis/jix131 28911043

[17] Won EJ, Choi MJ, Kim MN, Yong D, Lee WG, Uh Y, Kim TS, Byeon SA, Lee SY, Kim SH, Shin JH. 2021. Fluconazole-resistant Candida glabrata bloodstream isolates, South Korea, 2008-2018. Emerg Infect Dis 27:779–788. doi:10.3201/eid2703.203482 33624581 PMC7920659

[18] Patra S, Raney M, Pareek A, Kaur R. 2022. Epigenetic regulation of antifungal drug resistance. J Fungal 8:875. doi:10.3390/jof8080875 PMC9409733 36012862

[19] Ksiezopolska E, Schikora-Tamarit MÀ, Beyer R, Nunez-Rodriguez JC, Schüller C, Gabaldón T. 2021. Narrow mutational signatures drive acquisition of multidrug resistance in the fungal pathogen Candida glabrata. Curr Biol 31:5314–5326. doi:10.1016/j.cub.2021.09.084 34699784 PMC8660101

[20] Zhao C-R, You Z-L, Chen D-D, Hang J, Wang Z-B, Ji M, Wang L-X, Zhao P, Qiao J, Yun C-H, Bai L. 2023. Structure of a fungal 1,3-β-glucan synthase. Sci Adv 9:eadh7820. doi:10.1126/sciadv.adh7820 37703377 PMC10499315

[21] Bhakt P, Raney M, Kaur R. 2022. The SET-domain protein CgSet4 negatively regulates antifungal drug resistance via the ergosterol biosynthesis transcriptional regulator CgUpc2a. J Biol Chem 298:102485. doi:10.1016/j.jbc.2022.102485 36108742 PMC9576903

[22] Garcia-Rubio R, Jimenez-Ortigosa C, DeGregorio L, Quinteros C, Shor E, Perlin DS. 2021. Multifactorial role of mitochondria in echinocandin tolerance revealed by transcriptome analysis of drug-tolerant cells. MBio 12:e0195921. doi:10.1128/mBio.01959-21 34372698 PMC8406274

[23] O’Kane CJ, Weild R, M. Hyland E. 2020. Chromatin structure and drug resistance in Candida spp. JoF 6:121. doi:10.3390/jof6030121 32751495 PMC7559719

[24] Nikolov VN, Malavia D, Kubota T. 2022. SWI/SNF and the histone chaperone Rtt106 drive expression of the pleiotropic drug resistance network genes. Nat Commun 13:1968. doi:10.1038/s41467-022-29591-z 35413952 PMC9005695

[25] Sahu MS, Purushotham R, Kaur R. 2024. The Hog1 MAPK substrate governs Candida glabrata-epithelial cell adhesion via the histone H2A variant. PLoS Genet 20:e1011281. doi:10.1371/journal.pgen.1011281 38743788 PMC11125552

[26] Mc.Koy JF, Pleninger P, Wall L, Pramanik A, Martinez M, Moore CW. 1995. Genetic changes and bioassays in bleomycin- and phleomycin-treated cells, and their relationship to chromosomal breaks. Mutation Research/DNA Repair 336:19–27. doi:10.1016/0921-8777(94)00040-D 7528892

[27] Zhang M, Liang Y, Zhang X, Xu Y, Dai H, Xiao W. 2008. Deletion of yeast CWP genes enhances cell permeability to genotoxic agents. Toxicol Sci 103:68–76. doi:10.1093/toxsci/kfn034 18281714

[28] Tyler JK, Adams CR, Chen S-R, Kobayashi R, Kamakaka RT, Kadonaga JT. 1999. The RCAF complex mediates chromatin assembly during DNA replication and repair. Nature New Biol 402:555–560. doi:10.1038/990147 10591219

[29] Driscoll R, Hudson A, Jackson SP. 2007. Yeast Rtt109 promotes genome stability by acetylating histone H3 on lysine 56. Science 315:649–652. doi:10.1126/science.1135862 17272722 PMC3334813

[30] Rai MN, Balusu S, Gorityala N, Dandu L, Kaur R. 2012. Functional genomic analysis of Candida glabrata-macrophage interaction: role of chromatin remodeling in virulence. PLoS Pathog 8:e1002863. doi:10.1371/journal.ppat.1002863 22916016 PMC3420920

[31] Wurtele H, Tsao S, Lépine G, Mullick A, Tremblay J, Drogaris P, Lee EH, Thibault P, Verreault A, Raymond M. 2010. Modulation of histone H3 lysine 56 acetylation as an antifungal therapeutic strategy. Nat Med 16:774–780. doi:10.1038/nm.2175 20601951 PMC4108442

[32] Sutton A, Bucaria J, Osley MA, Sternglanz R. 2001. Yeast ASF1 protein is required for cell cycle regulation of histone gene transcription. Genetics 158:587–596. doi:10.1093/genetics/158.2.587 11404324 PMC1461693

[33] Denning DW. 2024. Global incidence and mortality of severe fungal disease. Lancet Infect Dis 24:e428–e438. doi:10.1016/S1473-3099(23)00692-8 38224705

[34] Prado F, Aguilera A. 2005. Partial depletion of histone H4 increases homologous recombination-mediated genetic instability. Mol Cell Biol 25:1526–1536. doi:10.1128/MCB.25.4.1526-1536.2005 15684401 PMC548009

[35] Bhagwat M, Nagar S, Kaur P, Mehta R, Vancurova I, Vancura A. 2021. Replication stress inhibits synthesis of histone mRNAs in yeast by removing Spt10p and Spt21p from the histone promoters. J Biol Chem 297:101246. doi:10.1016/j.jbc.2021.101246 34582893 PMC8551654

[36] Mei Q, Huang J, Chen W, Tang J, Xu C, Yu Q, Cheng Y, Ma L, Yu X, Li S. 2017. Regulation of DNA replication-coupled histone gene expression. Oncotarget 8:95005–95022. doi:10.18632/oncotarget.21887 29212286 PMC5706932

[37] Tscherner M, Stappler E, Hnisz D, Kuchler K. 2012. The histone acetyltransferase Hat1 facilitates DNA damage repair and morphogenesis in Candida albicans. Mol Microbiol 86:1197–1214. doi:10.1111/mmi.12051 23075292

[38] Lopes da Rosa J, Boyartchuk VL, Zhu LJ, Kaufman PD. 2010. Histone acetyltransferase Rtt109 is required for Candida albicans pathogenesis . Proc Natl Acad Sci USA 107:1594–1599. doi:10.1073/pnas.0912427107 20080646 PMC2824404

[39] Liu Z, Myers LC. 2017. Candida albicans Swi/Snf and mediator complexes differentially regulate Mrr1-induced MDR1 expression and fluconazole resistance . Antimicrob Agents Chemother 61:1–24. doi:10.1128/AAC.01344-17 PMC5655090 28807921

[40] Shivarathri R, Tscherner M, Zwolanek F, Singh NK, Chauhan N, Kuchler K. 2019. The fungal histone acetyl transferase Gcn5 controls virulence of the human pathogen Candida albicans through multiple pathways. Sci Rep 9:9445. doi:10.1038/s41598-019-45817-5 31263212 PMC6603162

[41] Yu S, Paderu P, Lee A, Eirekat S, Healey K, Chen L, Perlin DS, Zhao Y. 2022. Histone acetylation regulator Gcn5 mediates drug resistance and virulence of Candida glabrata. Microbiol Spectr 10:e0096322. doi:10.1128/spectrum.00963-22 35658596 PMC9241792

[42] Baker KM, Hoda S, Saha D, Gregor JB, Georgescu L, Serratore ND, Zhang Y, Cheng L, Lanman NA, Briggs SD. 2022. The Set1 Histone H3K4 methyltransferase contributes to azole susceptibility in a species-specific manner by differentially altering the expression of drug efflux pumps and the ergosterol gene pathway. Antimicrob Agents Chemother 66:e0225021. doi:10.1128/aac.02250-21 35471041 PMC9112889

[43] Moirangthem R, Kumar K, Kaur R. 2021. Two functionally redundant FK506-binding proteins regulate multidrug resistance gene expression and govern azole antifungal resistance. Antimicrob Agents Chemother 65:e02415-20. doi:10.1128/AAC.02415-20 33722894 PMC8316114

[44] Steen BR, Lian T, Zuyderduyn S, MacDonald WK, Marra M, Jones SJM, Kronstad JW. 2002. Temperature-regulated transcription in the pathogenic fungus Cryptococcus neoformans. Genome Res 12:1386–1400. doi:10.1101/gr.80202 12213776 PMC186651

[45] Wang M, Li J, Wang Y, Fu H, Qiu H, Li Y, Li M, Lu Y, Fu YV. 2023. Single-molecule study reveals Hmo1, not Hho1, promotes chromatin assembly in budding yeast. MBio 14:e0099323. doi:10.1128/mbio.00993-23 37432033 PMC10470511

[46] Georgieva M, Roguev A, Balashev K, Zlatanova J, Miloshev G. 2012. Hho1p, the linker histone of Saccharomyces cerevisiae, is important for the proper chromatin organization in vivo. Biochim Biophys Acta 1819:366–374. doi:10.1016/j.bbagrm.2011.12.003 22200500

[47] Bairwa G, Kaur R. 2011. A novel role for a glycosylphosphatidylinositol-anchored aspartyl protease, CgYps1, in the regulation of pH homeostasis in Candida glabrata. Mol Microbiol 79:900–913. doi:10.1111/j.1365-2958.2010.07496.x 21299646

