# Supplementary material for: Core histones govern echinocandin susceptibility in Candida glabrata
Source: Microbiol Spectr. 2025 Apr 30;13(6):e02399-24. doi: 10.1128/spectrum.02399-24 (PMC12131851; doi:10.1128/spectrum.02399-24)

**Core histones govern echinocandin susceptibility in *Candida glabrata***

Aditi Pareek^1,2^ and Rupinder Kaur^1,*^

^1^Laboratory of Fungal Pathogenesis, Centre for DNA Fingerprinting and Diagnostics, Hyderabad-500039, India

^2^Graduate studies, Manipal Academy of Higher Education, Manipal-576104, Karnataka, India

^*^For correspondence: Rupinder Kaur, BRIC-Centre for DNA Fingerprinting and Diagnostics (CDFD), Hyderabad, Telangana, India. Tel.: 91-40-27216137; Fax: 91-40-27216006; E-mail: [rkaur@cdfd.org.in](mailto:rkaur@cdfd.org.in).

**Supplemental Material**

The manuscript contains five supplementary figures (Figures S1-S5) and five supplementary tables (Tables S1-S5).

**Supplementary figure legends**

**Figure S1: Genomic organization of *CgHTA* and *CgHTB* loci**

1. Schematic illustration of histone H2A-encoding ORFs, *CgHTA1* and *CgHTA2*, and histone H2B-encoding ORFs, *CgHTB1* and *CgHTB2* in *C. glabrata. CgHTA1* and *CgHTB1* gene pair is located on chromosome K, while *CgHTA2* and *CgHTB2* gene pair is located on chromosome C. The schematic is not drawn to scale.
2. Multiple amino acid sequence alignment of histone H2A encoded by the two histone H2A ORFs, *CgHTA1* and *CgHTA2*. The Clustal Omega tool (<https://www.ebi.ac.uk/Tools/msa/clustalo/>) was used to align sequences retrieved from the Uniprot database (<https://www.uniprot.org/>) or CGD database (http://www.candidagenome.org/) for each protein.
3. Multiple amino acid sequence alignment of histone H2B encoded by the two histone H2B ORFs, *CgHTB1* and *CgHTB2*. The Clustal Omega tool (<https://www.ebi.ac.uk/Tools/msa/clustalo/>) was used to align sequences retrieved from the Uniprot database (<https://www.uniprot.org/>) or CGD database (http://www.candidagenome.org/) for each protein.

**Figure S2: Dose response curves illustrating the increased susceptibility of core histone mutants towards caspofungin.** Overnight YPD medium-grown cultures (1 x 10^4^ cells) of indicated *C. glabrata* strains were inoculated in the glucose (2%-) and MOPS (0.165 M; pH 7.0)-containing RPMI medium in the absence or presence of caspofungin (CSP; 1 to 1024 ng/ml) in a 96-well plate. After 24 h incubation at 37°C, the culture absorbance was recorded at 530 nm. Data (mean ± SEM; n = 3) represent a decrease in absorbance of the caspofungin-treated cultures, compared to the absorbance of untreated cultures of respective strains (considered as 100). Statistically-significant differences in % decrease in the absorbance between wild-type and mutants were observed as follows: *Cghta2Δ* (*p* ≤ 0.0005 at 256 ng/ml caspofungin), *Cghtb2Δ* (*p* ≤ 0.0336 and *p* ≤ 0.0001 at 128 and 256 ng/ml caspofungin, respectively), *Cghht2Δ3Δ* (*p* ≤ 0.0067 and *p* ≤ 0.0002 at 128 and 256 ng/ml caspofungin, respectively) and *Cghhf2Δ3Δ* (*p* ≤ 0.0156 and *p* ≤ 0.0001 at 128 and 256 ng/ml caspofungin, respectively).

**Figure S3: Heat map illustrating growth of *C. glabrata* strains in indicated medium.** Optical density (OD_600_) of overnight YPD medium-grown cells was adjusted to 1.0, followed by four 10-fold serial dilutions in PBS. 3 µl of each dilution was plated on YPD medium in the presence or absence of the stressor. Images were captured after 1-2 days of growth at 30^ο^C, and growth of each mutant strain was scored with respect to the *wt* growth in the same medium (*wt* growth in each medium was assigned a score of 0). Heat map was generated using the matrix2png web application interface (<https://matrix2png.msl.ubc.ca/bin/matrix2png.cgi>), and represents the comparative growth analysis of the indicated histone mutant as compared to that of the *wt* strain in the same medium. Stressors used were β-mercaptoethanol (β-ME; 8 mM), Sodium dodecyl sulfate (SDS; 0.05%), Congo red (CR;2 mg/ml), Calcofluor white (CFW;2 mg/ml), Menadiaone (MD;100 µM), Hydrogen peroxide (H_2_O_2_; 40 µM), Hydroxyurea (HU; 100 mM), Methyl methanesulfonate (MMS; 0.04%), Phleomycin (Phleo; 10 µM), Fluconazole (FLC; 16 µg/ml), Amphotericin B-(AMB; 1.0 µg/ml) and Caspofungin (CSP;150 ng/ml).

**Figure S4: Histone mutants are not susceptible to Zymolyase digestion.** YPD medium-grown, log-phase cells (2.0 OD_600_ cells) of indicated strains were incubated with 50 μg/ml zymolyase for 8 h. Absorbance was measured for every 10 min interval at 600 nm wavelength. Data (mean ± SD; n = 2) represent as percent change in the absorbance of each strain, compared to the respective 0 h absorbance (taken as 100).

**Figure S5: Serial dilution spotting assay illustrating vitamin C-mediated rescue of increased caspofungin susceptibility of indicated mutants.** Caspofungin (CSP) was added to a final concentration of 125 ng/ml. VitC, Vitamin C.

**Supplementary Tables (Tables S1 to S5)**

**Table S1: List of canonical histone-encoding ORFs in *C. glabrata*.**

**Table S2: List of strains used in the study.**

**Table S3: List of plasmids used in the study.**

**Table S4: List of primers used in the study.**

**Table S5: List of antibodies (A), chemicals, commercial kits and software (B) used in the study**


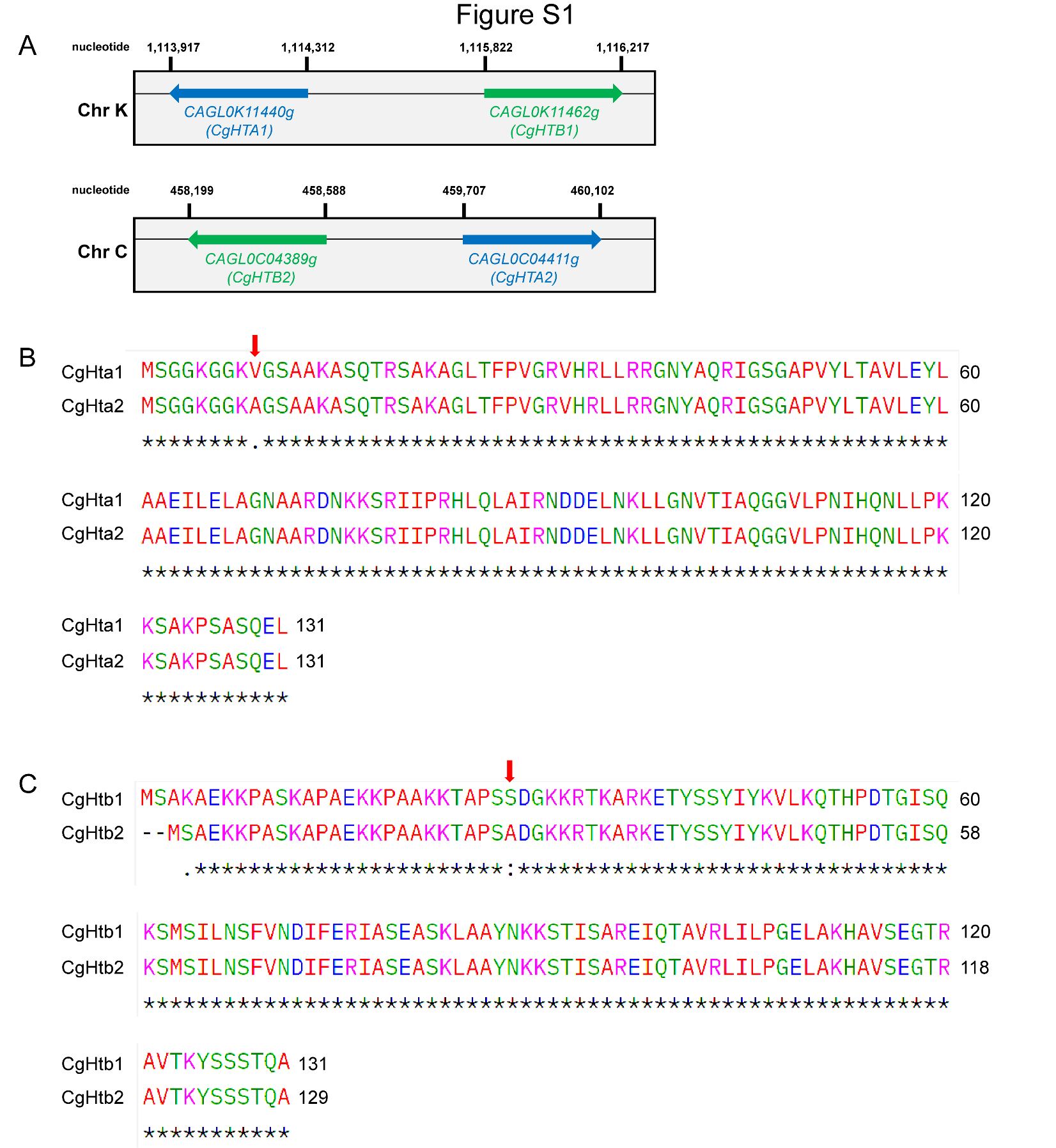


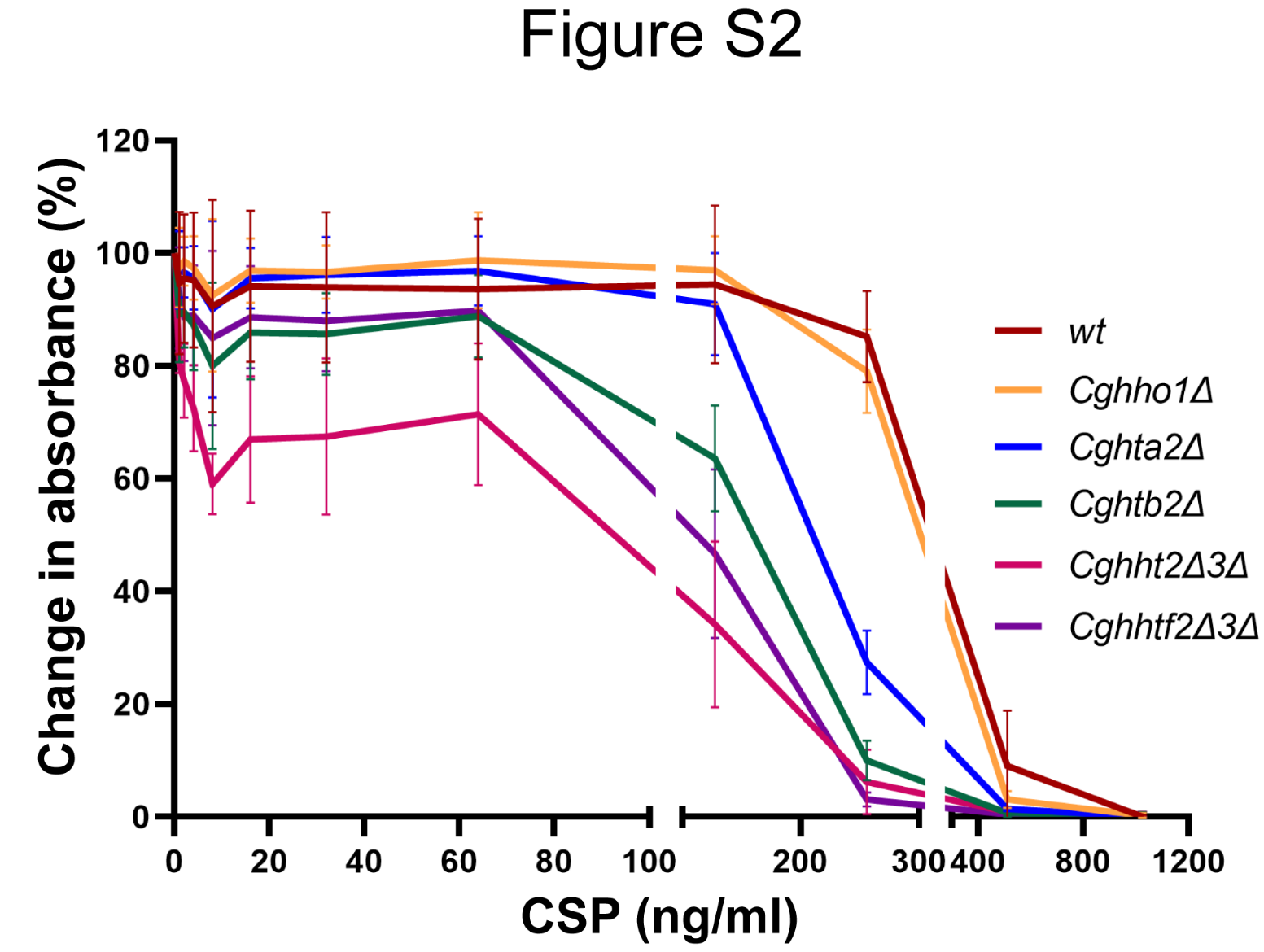


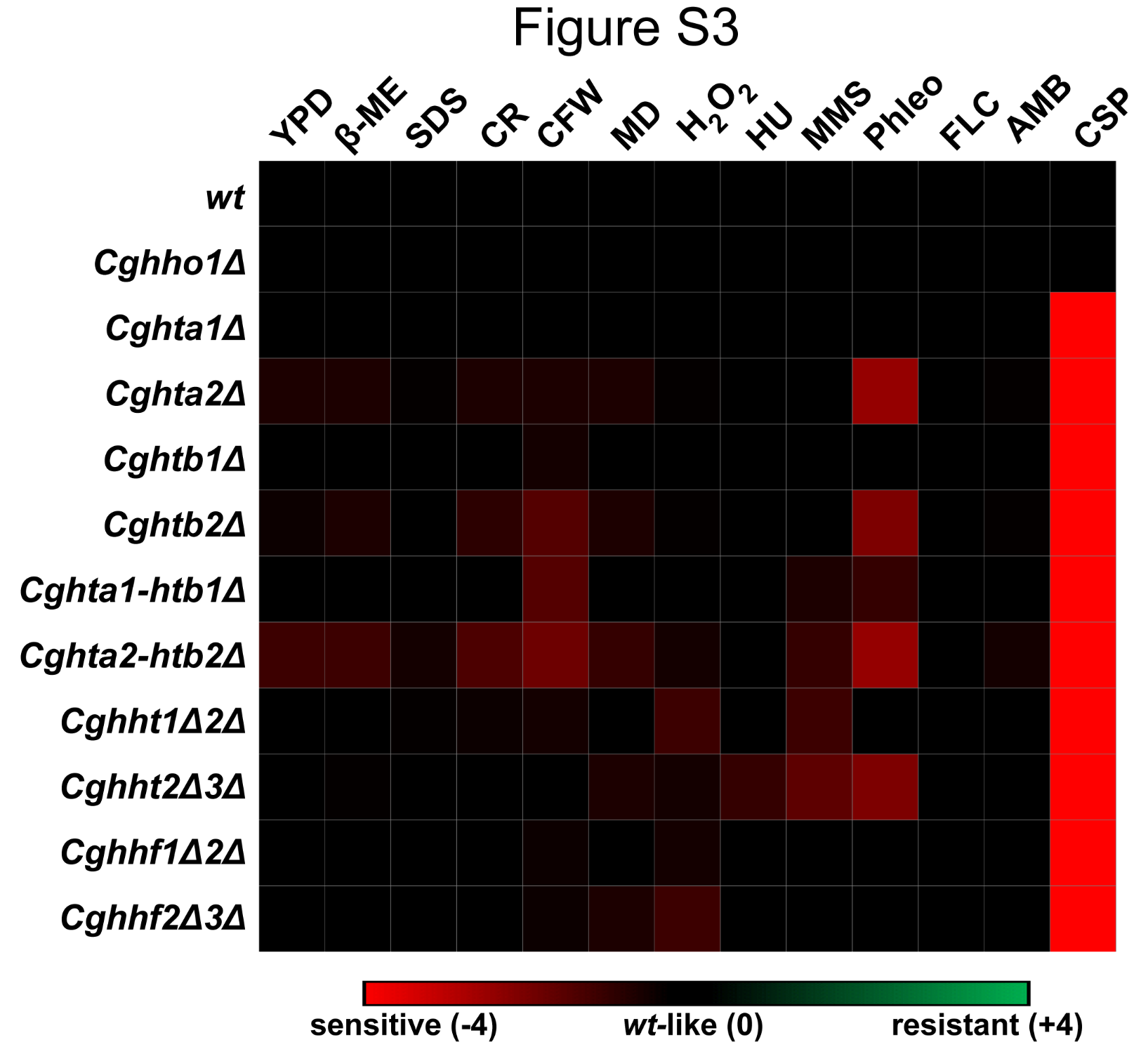


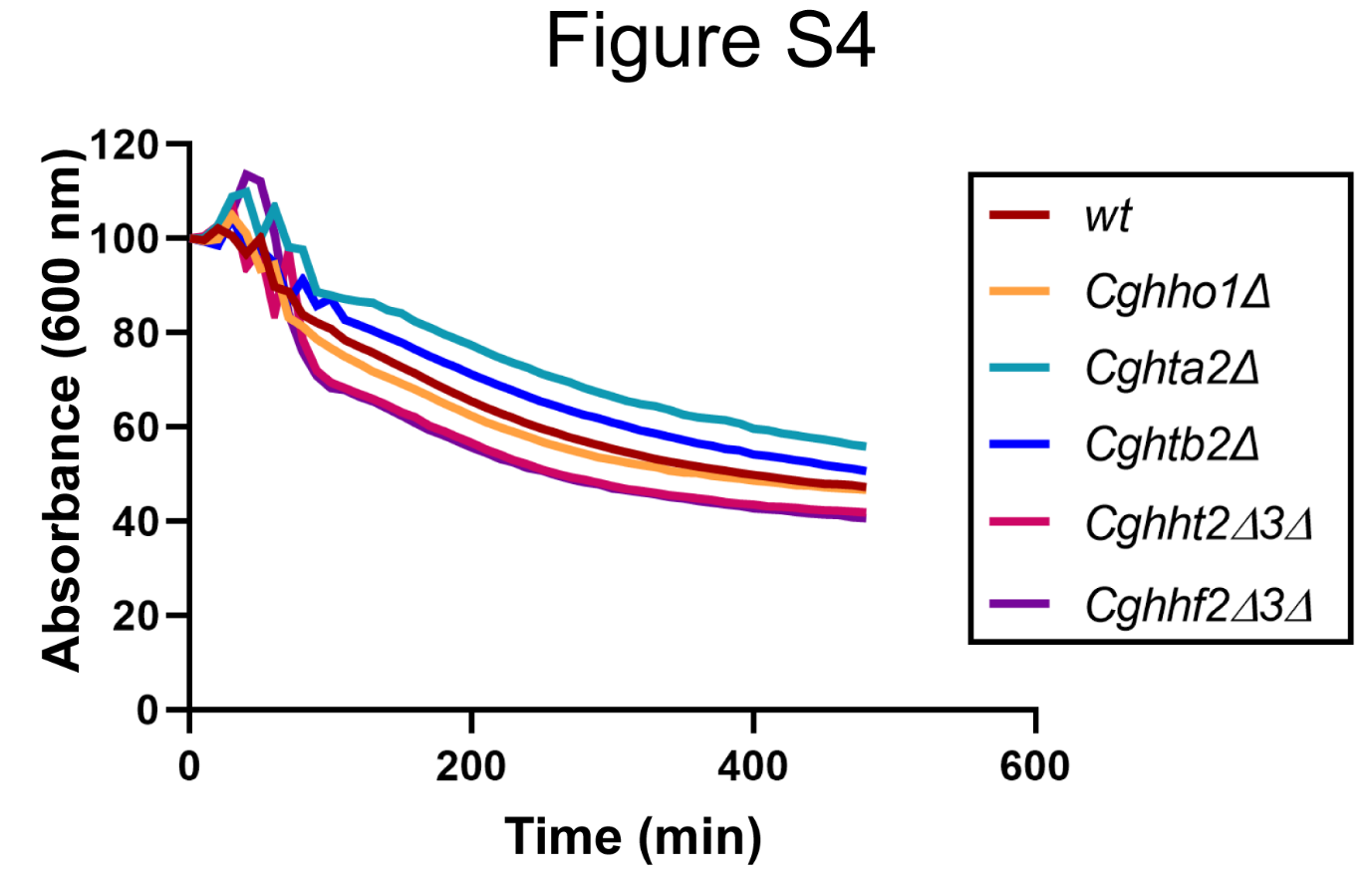


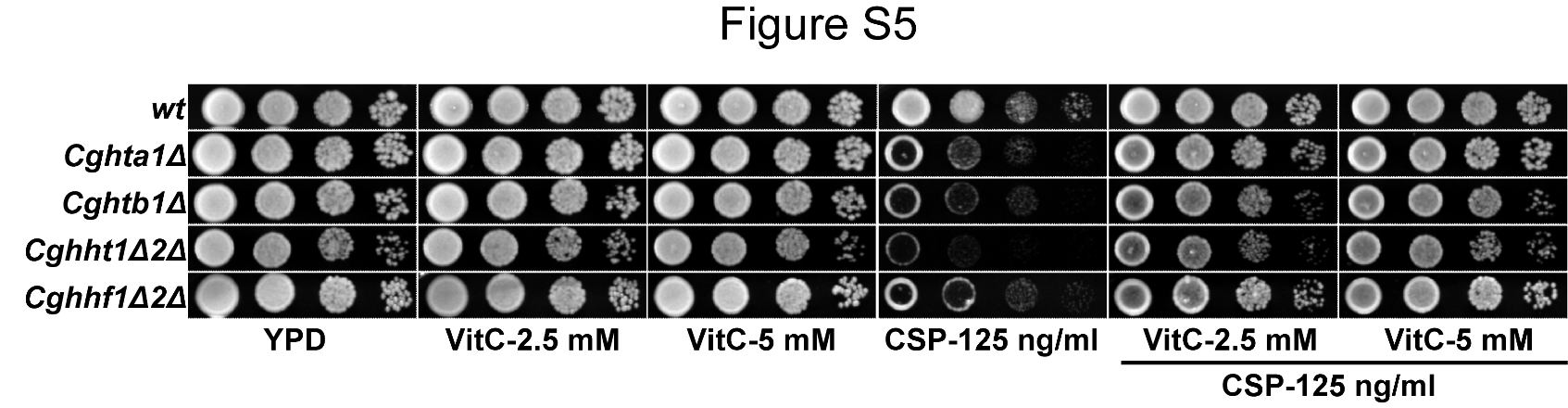

Supplement: Supplemental material — Fig. S1 to S5; Supplemental legends. [file spectrum.02399-24-s0001.docx]
